# Nanoparticle-Mediated Interface Engineering for Uniform, Reproducible Electron Transport Layers in Scalable Perovskite Solar Cells

**DOI:** 10.1021/acsami.5c24295

**Published:** 2026-02-18

**Authors:** Charlie Henderson, Adriano S. Marques, Izabela S. Bicalho, Lucy J. F. Hart, Amy Monahan, Katherine Stewart, Koki Asano, Tianhao Lan, Martin Vacha, Molly M. Stevens, Piers R. F. Barnes, Diego Bagnis, Ji-Seon Kim

**Affiliations:** † Department of Physics and Centre for Processable Electronics, 4615 Imperial College London, London SW7 2AZ, U.K.; ‡ Oninn Centro de Inovações, 31035536 Belo Horizonte, MG, Brazil; § Department of Chemistry and Centre for Processable Electronics, 4615 Imperial College London, London W12 0BZ, U.K.; ∥ Department of Materials, Department of Bioengineering and Institute of Biomedical Engineering, 4615 Imperial College London, London SW7 2AZ, U.K.; ⊥ Department of Physiology, Anatomy and Genetics, Department of Engineering Science, Kavli Institute for Nanoscience Discovery, 6396 University of Oxford, Oxford OX1 3QU, U.K.; # Department of Materials Science and Engineering, School of Materials and Chemical Technology, 693022 Institute of Science Tokyo, Tokyo 152-8552, Japan; ¶ Department of Chemistry, University of Oxford, South Parks Road, Oxford OX1 3QZ, U.K.; ○ Department of Physics, Ewha Womans University, Seoul 03760, Republic of Korea

**Keywords:** photovoltaics, perovskites, scalable processing, interface engineering, interfacial charge extraction

## Abstract

As lab-scale perovskite solar cells (PSCs) approach their efficiency limits, reproducing this performance in large-area, manufacturable devices remains challenging. Here, we show that printing interlayers of metal oxide nanoparticles, specifically Al_2_O_3_ and SnO_2_, can systematically control the morphology and interfacial energetics of solution-processed PC_61_BM electron transport layers (ETLs) in flexible roll-to-roll printed PSCs. These nanoparticle interlayers enhance ETL uniformity, reduce pinholes, and increase shunt resistance, improving power conversion efficiencies (PCEs) and reducing device failure rates by 50%. Through a combination of systematic device characterization, morphological, spectroscopic and energetic analysis, coupled with drift-diffusion simulations, the distinct roles of insulating (Al_2_O_3_) and semiconducting (SnO_2_) nanoparticle interlayers in mediating carrier extraction and recombination are elucidated. Al_2_O_3_ suppresses interfacial recombination and improves device reproducibility, albeit with some penalty in short-circuit current, whereas SnO_2_ enhances electronic coupling and charge extraction, delivering a champion PCE of 11.0% (active area: 0.5 cm^2^). Incorporating SnO_2_ interlayers into larger-area modules (active area: 7.2 cm^2^) further demonstrates the robustness of this strategy under manufacturing-relevant conditions. Together, these results provide an important framework for nanoparticle-mediated interface engineering and establish a simple, effective, and scalable route to improving both performance and yield in printed large-area PSCs.

## Graphical abstract

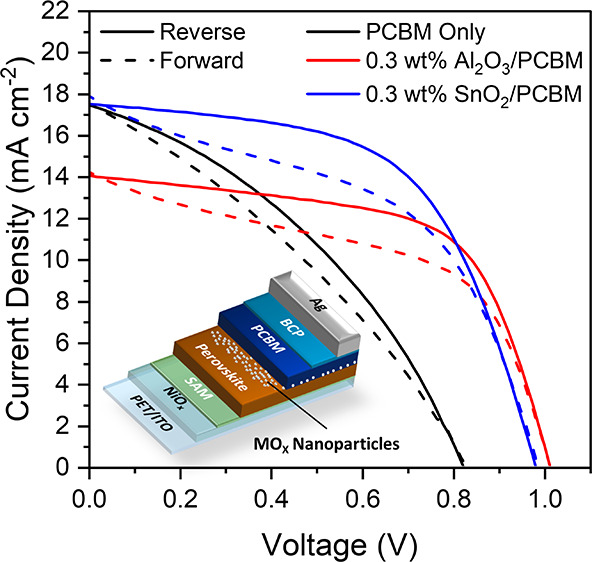

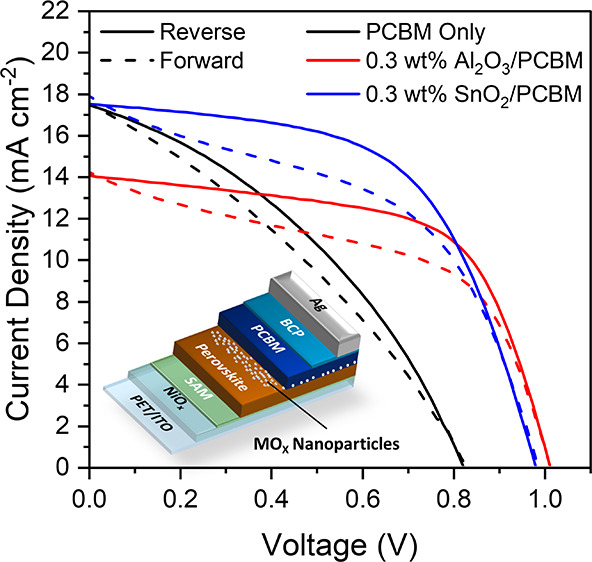

## Introduction

The power conversion efficiency (PCE) of research scale metal halide perovskite (MHP) solar cells now exceeds 26%,^1^ comparable to the best silicon based modules. However, significant challenges remain in reproducing this performance at scales relevant for power generation. Compared to traditional crystalline inorganic semiconductors, perovskites are more tolerant to mechanical deformation, making them attractive for continuous roll-to-roll (R2R) processing.^2,3^

Perovskite films can be formed by either solution processing, where a wet film containing the precursor materials is coated onto a substrate, or by evaporation, where precursor materials are evaporated under ultrahigh vacuum onto a substrate where the perovskite forms.^4^ Both are methods are being targeted for industrial scale up, however, to the best of our knowledge, only solution processing has been successfully demonstrated in a continuous R2R process.^5^ High capital expenditure to procure equipment and the relatively early stage of evaporated perovskite research (as compared to solution processing) currently makes this technique less attractive to industry. In this work we focus on solution processed perovskite solar cells.

Presently, most MHP research is focused on spin-casting films which, in an academic laboratory, is a simple process resulting in high quality, reproducible films. However, substrate size constraints, high material wastage, and incompatibility with continuous processing makes spin coating unattractive for commercial scale up.

Sequential printing of the required device layers is a significant challenge for upscaling as methods used for small, spin coated devices often do not translate directly to meniscus assisted techniques such as blade or slot-die coating.^6^ An example is the printing of PC_61_BM electron transport layers. PC_61_BM coating on perovskite is facile using a spin coater and rarely requires extensive optimization for regular Pb based perovskites. However, when moving to larger areas using blade or slot-die coating, preventing pinhole formation and dewetting becomes a significant challenge.^7^

The deposition of high-quality blade coated perovskite films can be assisted by depositing noncontinuous metal oxide nanoparticle seeding layers. The nanoparticles seed the crystallization of a uniform perovskite film on top of hole transport materials (HTMs) with poor wettability in both spin, blade, and spray coated devices.^8–11^ Recent work by Jin et al. demonstrated that the quality of spin-cast PC_61_BM films deposited on Sn–Pb narrow band gap perovskite was improved by prior application of a thin layer of spin-cast Al_2_O_3_ nanoparticles on the perovskite surface.^12^ They found that this layer enabled the PC_61_BM to conformally coat the perovskite surface, reducing shunts and improving device performance. Uddin et al. and Li et al. have also previously demonstrated that additives in the PC_61_BM solution result in improved performance in blade coated perovskite devices.^7,13^

The goal of this work is to improve the printing of fullerene electron transport layers (ETLs) from solution, toward full R2R solution processing of perovskite solar cells. To this end we apply blade coated nanoparticle layers of two metal oxides, alumina (Al_2_O_3_) and tin oxide (SnO_2_), between the perovskite and PC_61_BM. The former is an electronic insulator, while the latter is an n-type semiconductor. Both result in improved uniformity of the PC_61_BM film, increasing the reproducibility and performance of the fabricated solar cells. We investigate the impact of nanoparticle type on the modification of the perovskite/PC_6__1_BM interface, demonstrating that nanoparticle electronic structure plays a key role in governing interfacial charge transfer. Finally, we demonstrate the utility of this approach for the fabrication of large area devices by applying SnO_2_ nanoparticles in minimodules.

## Results and Discussion

### Comparison of Evaporated and Solution-Processed Electron Transport Layers

Typical p-i-n perovskite solar cells incorporate fullerene-based electron transport layers, usually evaporated C_60_ or solution processed phenyl-C_61_-butyric acid methyl ester (PC_61_BM). We fabricated flexible p-i-n devices with the structure: polyethylene terephthalate (PET)/ITO/NiO_*x*_/MeO-2PACz/perovskite/*fullerene*/bathocuproine (BCP)/Ag, where ‘fullerene’ is either solution processed PC_61_BM or evaporated C_60_. In all devices the NiO_*x*_, MeO-2PACz and perovskite layers were deposited sequentially via slot die coating in a roll-to-roll process outlined in Figure 1a.^14^ The PC_61_BM/BCP layers were deposited sequentially by blade coating and the C_60_/BCP layers were deposited sequentially by thermal evaporation. All devices had thermally evaporated Ag cathodes. All printed layers were processed in ambient air in a cleanroom environment.

**Figure 1:**
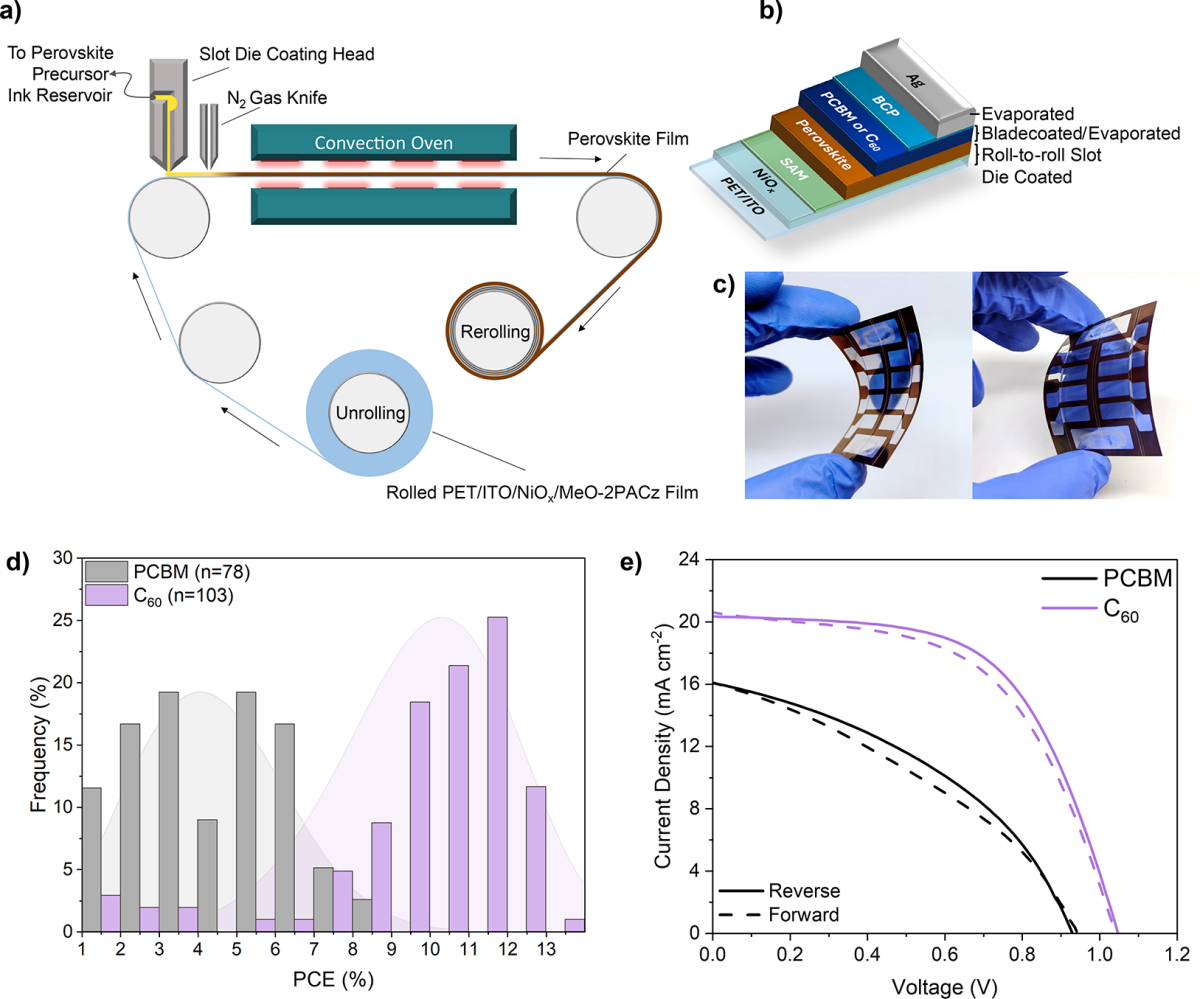
(a) Simplified diagram of the roll-to-roll printing process used to produce the perovskite films in this work; (b) reference device stack; (c) photographs showing example flexible devices; (d) histogram showing the PCE distributions of PSCs utilizing PC_61_BM (black) and C_60_ (purple) ETLs (devices with PCE < 1% excluded); and (e) example JV curves of the PSCs shown in Figure 1d.

Devices with blade coated PC_61_BM ETLs exhibited exceptionally low performance and poor reproducibility compared to devices with evaporated C_60_ ETLs (Figure 1d,e). The performance drop in the PC_61_BM devices is driven by reductions in the open circuit photovoltage (*V*_OC_), the fill factor (FF) and short circuit photocurrent density (*J*_SC_), with the shunt and series resistance (*R*_shunt_ and *R*_series_) also decreasing and increasing respectively (Figure S1, Table S1). Of the 136 reference PC_61_BM devices fabricated for this study, 38% had PCEs below 1% (our threshold for a ‘failed’ device), compared to just 4% of the C_60_ based devices.

### PC_61_BM Film Formation Dictated by the Presence of Nanoparticle Interlayers

A recent report by Jin et al. showed that inserting a discontinuous, spin-coated Al_2_O_3_ nanoparticle interlayer between mixed Sn/Pb perovskites and PC_6__1_BM improves PC_6__1_BM coverage and device performance.^12^ However, printed metal-oxide nanoparticle interlayers for flexible, roll-to-roll perovskite solar cells remain unaddressed. Here, we transform this concept to scalable processing.

We blade coated both insulating Al_2_O_3_ and n-type semiconducting SnO_2_ nanoparticles at a range of concentrations in isopropyl alcohol (IPA) followed sequentially by the PC_61_BM layer in o-xylene. o-Xylene was selected due to its significantly lower toxicity compared to chlorobenzene, the most common solvent used for PC_61_BM processing in PSCs. The reduced toxicity makes o-xylene more attractive for scale up manufacturing due to significantly reduced work place exposure limits.^15,16^

The Al_2_O_3_ and SnO_2_ nanoparticles form a noncontinuous layer on the perovskite, with the device optimized concentration of 0.3 wt % resulting in approximately 20% surface coverage of the perovskite film as quantified by scanning electron microscopy (SEM) (Figures S2 and S3). The SEM also demonstrates the functional difference between the insulating Al_2_O_3_ and the semiconducting SnO_2_. The increased brightness of the Al_2_O_3_ nanoparticles compared to the SnO_2_ nanoparticles is a result of charging of the Al_2_O_3_ nanoparticles by incident electrons from the field emission gun. Due to the insulating nature of Al_2_O_3_, incident electrons accumulate in the nanoparticles, leading to scattering of successive incident electrons.^17^ In contrast, the SnO_2_ particles remain similarly bright as the underlying perovskite due to their semiconducting nature allowing them to redistribute charge to the perovskite surface and subsequently to ground.

The impact of nanoparticles on PC_61_BM film formation is shown in Figure 2. SEM images of the reference perovskite/PC_61_BM films have large bright areas which are several microns across; image analysis shows these regions cover approximately 7% of the total film (Figure S4). We note that these areas show the morphology of the underlying perovskite film more clearly and with a higher brightness. In contrast both the Al_2_O_3_ and SnO_2_ modified films are more uniform under SEM, with no large bright regions. Further SEM images of the films at different magnifications are shown in Figure S5. To understand the chemical makeup of these regions we applied Raman mapping (Figures 2d–f and S6). By mapping the intensity of the characteristic PC_61_BM peak at 1465 cm^–1^ we find that the bright areas in the SEM image are vacancies in the PC_61_BM film.^18,19^ In contrast, the nanoparticle/PC_61_BM films have no vacancies visible in either the SEM images or Raman maps. The effect of these vacancies is evident to the naked eye, with the PC_61_BM only films appearing lighter in color, less uniform and slightly cloudy compared to the nanoparticle/PC_61_BM films which are uniformly the characteristic brown color of PC_61_BM (Figure 2g–i).

**Figure 2:**
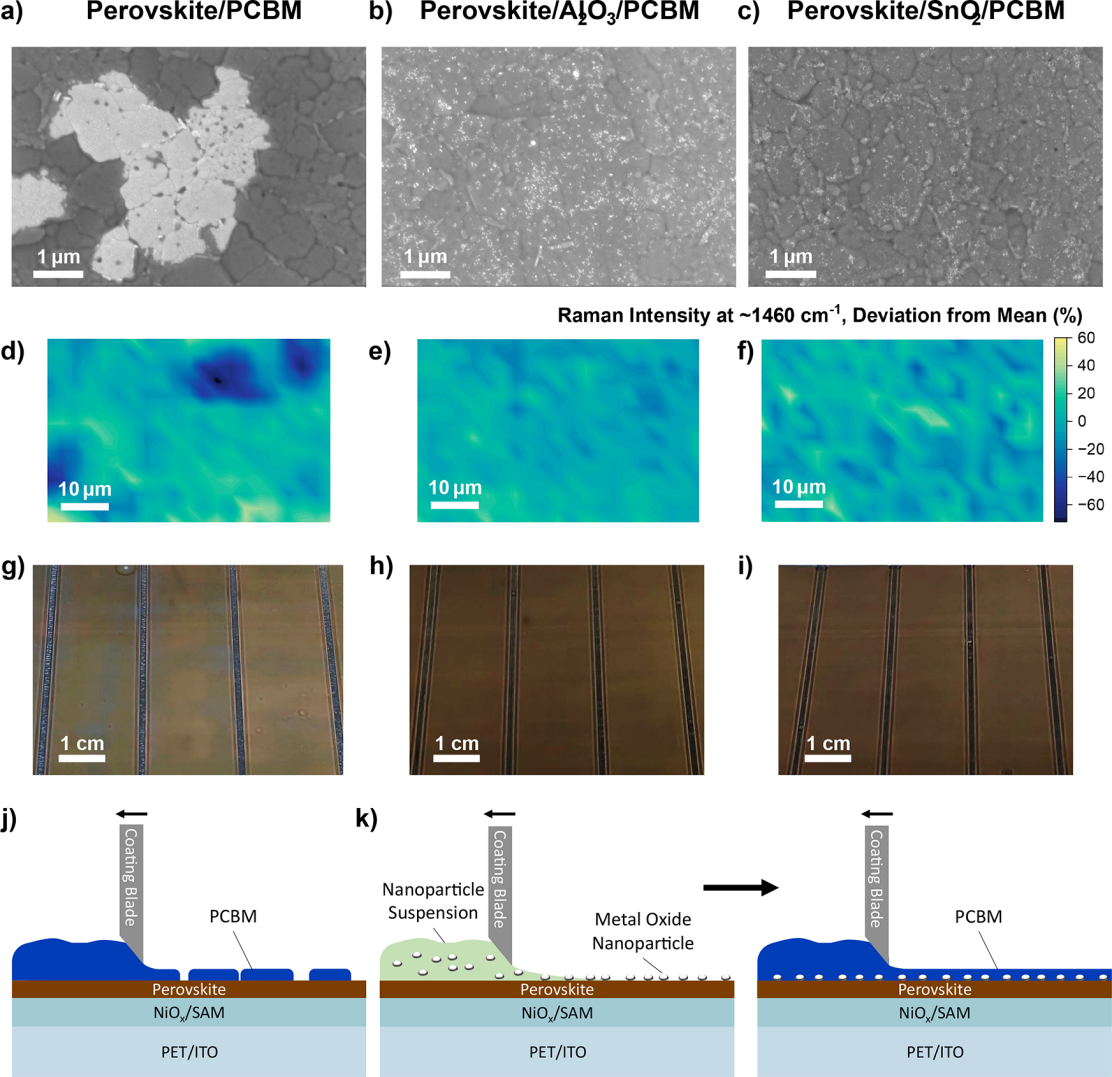
Impact of nanoparticles on the morphology of printed PC_61_BM films. All images were collected on films with the base structure of PET/NiO_*x*_/Me-2PACz/Perovskite. (a–c) SEM images of Perovskite/PC_61_BM, Perovskite/Al_2_O_3_ NPs/PC_61_BM, and Perovskite/SnO_2_ NPs/PC_61_BM films. (d–f) Maps of the intensity of the characteristic PC_61_BM Raman peak at ∼1460 cm^–1^ of Perovskite/PC_61_BM, Perovskite/Al_2_O_3_ NPs/PC_61_BM, and Perovskite/SnO_2_ NPs/PC_61_BM films. (g–i) Photographs of Perovskite/PC_61_BM, Perovskite/Al_2_O_3_ NPs/PC_61_BM, and Perovskite/SnO_2_ NPs/PC_61_BM films. Diagrams outlining the impact on PC_61_BM film coating without (j) and with (k) a metal oxide nanoparticle interlayer.

The surface morphology of the films was investigated using atomic force microscopy (AFM) (Figure S7). Unlike the work of Jin et al. we saw no significant change in the roughness of either the perovskite or perovskite/PC_61_BM films with and without nanoparticles present.^12^ This suggests that the roughness of the surface of the perovskite films is not the cause of the poor coating of PC_61_BM on the reference perovskite films.

### Impact of Metal Oxide Nanoparticle Interlayers on PSC Performance

To understand the impact of the improved PC_61_BM film morphology we applied the nanoparticle interlayers in PSCs of the structure (PET)/ITO/NiO_*x*_/MeO-2PACz/perovskite**/**Metal Oxide Nanoparticles/PC_61_BM/BCP/Ag. The results of the optimized concentration (0.3 wt % Al_2_O_3_ or SnO_2_ in IPA) are summarized in Figure 3.

**Figure 3:**
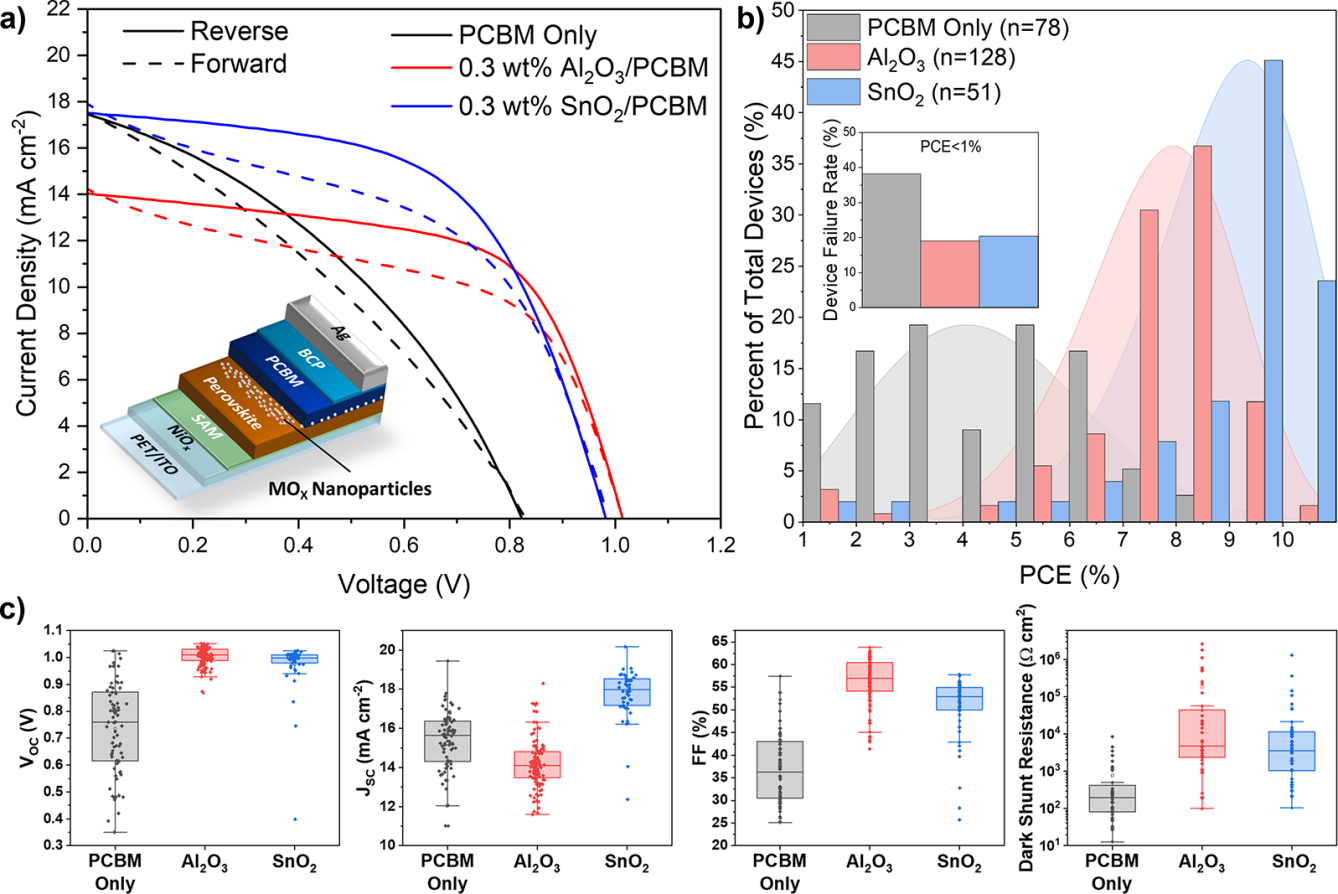
Impact of metal oxide nanoparticle interlayers on device performance. (a) JV curves of representative devices with and without 0.3 wt % nanoparticle interlayers. The inset diagram shows the complete device structure. (b) Histogram showing the PCE distribution of optimized devices with PCE ≥ 1%. The inset chart shows the percentage of devices of each type with PCE < 1%. (c) Statistics of selected performance parameters for each optimized device type.

Upon addition of Al_2_O_3_ nanoparticles we observe an increase in the PCE, FF, and *R*_shunt_, as well as a significant reduction in device-to-device *V*_OC_ variation. Additionally, we observe a substantial reduction in the proportion of failed devices (PCE < 1%) from 38% in the reference devices to less than 18% in the Al_2_O_3_ modified devices. Results of experiments to optimize the coating parameters and concentration of the Al_2_O_3_ nanoparticle suspension are shown in Figure S8. As the concentration of the Al_2_O_3_ solution increases from 0.15 wt % to the optimum 0.3 wt % we observe an increase in device PCE, FF, *V*_OC_ and *R*_shunt_. The addition of Al_2_O_3_ also results in decrease in device *J*_SC_ from an average of 15.3 ± 1.5 mA cm^–2^ in the reference devices to 14.0 ± 1.7 mA cm^–2^ in the optimized Al_2_O_3_ devices (Table S1). As the Al_2_O_3_ interlayer is noncontinuous (Figure S2), by increasing the concentration of the Al_2_O_3_ solution we are increasing the surface coverage of the nanoparticles. Al_2_O_3_ is an insulator which is not expected to mediate charge extraction from the perovskite,^20,21^ and therefore the decreased current in the devices with increased Al_2_O_3_ concentration may suggest that the Al_2_O_3_ nanoparticles block efficient charge extraction from the perovskite to the PC_61_BM ETL. This is confirmed by the presence of significant ‘s-shape’ kink in the JV of the devices with a 0.62 wt % Al_2_O_3_ interlayer (Figure S8), which is indicative of an extraction barrier at the ETL resulting in an imbalance of electron and hole extraction rates.^22–24^

To explore the effect of the electronic properties of the metal oxide nanoparticles on device performance we replaced the Al_2_O_3_ nanoparticle interlayer with SnO_2_ nanoparticles. SnO_2_ is an n-type semiconductor which is commonly applied as an ETM in perovskite solar cells.^25^ Figure 3 compares the device performance characteristics of devices incorporating blade coated layers of Al_2_O_3_ or SnO_2_ nanoparticles deposited from a 0.3 wt % solution. An increase in mean *J*_SC_ values of the SnO_2_ devices (17.8 ± 1.2 mA cm^–2^) versus the reference and Al_2_O_3_ devices is observed, with minimal losses in both FF and *V*_OC_. This leads to a significant increase in mean device PCE, with SnO_2_ based devices achieving 8.95 ± 1.87% PCE, compared to 7.64 ± 1.65% for the Al_2_O_3_-based devices and 4.39 ± 1.90% for the PC_61_BM only reference devices (Table S1). Additionally, the proportion of failed devices (PCE < 1%) is unchanged when Al_2_O_3_ is replaced with SnO_2_, although device to device variation is slightly increased. The performance of the SnO_2_ based devices is less dependent on the nanoparticle concentration compared to the Al_2_O_3_-based devices (Figure S9). As the concentration of SnO_2_ nanoparticles increases from 0.15 wt % there is an increase in *V*_OC_, *J*_SC_ and FF. Both *V*_OC_ and FF reach their maximum values at 0.3 wt % of SnO_2_ (the same concentration as for the Al_2_O_3_). However, the *J*_SC_ of the devices increases steadily to a maximum of 19.9 ± 0.8 mA cm^–2^ at the maximum tested concentration of 1.85 wt %. This suggests that, unlike the Al_2_O_3_, the SnO_2_ is active in extracting charges from the perovskite. Despite this high current, the drop in FF and *V*_OC_ at high SnO_2_ concentrations means that the devices with the highest efficiency are fabricated using 0.3 wt % of SnO_2_.

The performance of PC_61_BM only devices are limited by low shunt resistances, with most devices exhibiting shunt resistances below 1000 Ω cm^2^ with a median value of 194 Ω cm^2^ (Figure 3c). In contrast the median dark shunt resistances for devices with optimized Al_2_O_3_ and SnO_2_ interlayers are 4803 and 3605 Ω cm^2^ respectively. This observation is consistent with the large vacancies in the PC_61_BM film observed in the SEM images. By preventing the formation of these vacancies, the Al_2_O_3_ and SnO_2_ nanoparticle interlayers prevent direct contact between the perovskite and the Ag cathode – increasing the shunt resistance and therefore device performance.

To assess the stability of the devices under study, encapsulated Al_2_O_3_ and SnO_2_ nanoparticle modified devices were exposed to 1 Sun illumination in air under maximum power point tracking. The Al_2_O_3_ and SnO_2_ devices exhibited T_80_ of 112 and 400 h respectively (Figure S10).

We finally confirmed that the improvements in device performance described are a result of the presence of the metal oxide nanoparticles, and not because of the IPA solvent they are suspended in. We fabricated devices which were treated with an IPA ‘rinse’, where neat IPA was blade coated across the perovskite prior to PC_61_BM deposition using the same coating parameters as for the nanoparticles. We observed no difference in the performance of the reference devices and those treated with IPA, confirming that is the presence of the metal oxide nanoparticles, not their method of deposition, which is responsible for the observed improvement in performance (Figure S11).

### Role of Nanoparticles in Charge Carrier Recombination and Extraction

External quantum efficiency (EQE) spectra of the reference and optimized nanoparticle interlayer devices are shown in Figure S12. All devices have a similar response onset, with band gap analysis (Figure S13) confirming a photovoltaic bandgap of 1.58 eV.^26,27^ Integrating the EQE spectra to find the total current produced by the devices gives the same trend as the *J*_SC_ under 1 Sun illumination. By normalizing the spectra to their intensity at 400 nm the shape of the EQE responses can be compared. The normalized EQE response of both the Al_2_O_3_ and SnO_2_ devices are very similar in shape, suggesting that the difference in current between the Al_2_O_3_ and SnO_2_ devices is due to more efficient extraction of photogenerated charges in the SnO_2_ device. The shape of the reference PC_61_BM EQE spectrum is also similar to the nanoparticle modified devices at wavelengths <530 nm; however, at longer wavelengths a significant relative reduction in EQE is observed. Optical absorption depth in perovskite films is wavelength dependent, with short wavelength photons more likely to be absorbed close to the film’s surface and longer wavelength photons absorbed deeper into the film (i.e., closer to the perovskite/ETL interface).^28,29^ The reference device’s reduced EQE in the longer wavelength region is therefore consistent with increased recombination of photogenerated charges deeper in the film. Due to the incomplete coverage of the PC_61_BM film, this is likely caused by direct contact between the perovskite and the Ag cathode.

We applied photoluminescence (PL) microscopy to partial device stacks (without BCP or Ag) to understand the nanoscale photophysical properties of the films, and local charge extraction and recombination phenomena. Spatially mapping the PL intensity and spectra of the perovskite/PC_61_BM only film reveals wide distribution in PL intensity (Figure S14). This is consistent with the incomplete surface coverage of the perovskite in the PC_61_BM only sample leading to a reduction in PC_61_BM/perovskite interfacial area and consequently less surface recombination/PL quenching. Addition of the nanoparticle interlayers reduces both the baseline intensity and spatial heterogeneity of the PL spectra, consistent with more complete surface coverage of the perovskite by the electron extraction layers - resulting in greater quenching of PL. The distribution of the PL peak positions (likely a result of local differences in halide composition in the perovskite) is unchanged by addition of the nanoparticle interlayers and the ETL. This confirms that the observations are a result of variation in interfacial charge extraction efficiency, rather than perovskite composition.

Transient PL decays of perovskite/PC_61_BM samples with and without nanoparticle modification are given in Figure S15a. All three samples show a similar fast decay at early times (τ_1_ ≈ 1 ns), followed by a slower decay at longer times (τ_2_), as summarized in Figure S15b. The τ_2_ decay is shortest for the SnO_2_ nanoparticle modified sample (22.9 ns at 5 × 10^5^ Hz laser repetition rate) followed by the Al_2_O_3_ modified sample (25.0 ns) then the reference PC_61_BM only sample (26.2 ns). When interpreting the PL decay of perovskite films in contact with charge transport layers, the slower decay component (τ_2_) is typically associated with the lifetime of carriers that are not rapidly trapped or recombined at the interface and can therefore be quenched by charge transfer to the fullerene layer.^30^ Here we interpret the faster decays in the SnO_2_ samples as evidence for faster, more efficient, extraction of charges – consistent with the broader set of data presented. The slower decay in the reference sample is likely due to the incomplete coverage of the perovskite by the PC_61_BM resulting in reduced quenching of the PL. Importantly these differences in decay rate are independent of the repetition rate of the laser used, suggesting that the relative extraction rates are unaffected by state filling or carrier density.^31,32^ We also note that as the samples were illuminated through the PC_61_BM (meaning generation will occur close to the perovskite/ETL interface) the influence of carrier diffusion through the bulk perovskite upon the PL lifetimes will be minimal.^30^

To better understand the effect of the nanoparticles on charge extraction and recombination, we model the devices’ JV curves using Driftfusion,^33^ a drift-diffusion software designed to simulate ordered semiconductor devices containing mobile ionic charge. Details of the simulation parameters are given in Tables S2 and S3 and the protocol is given in the Methods section. We note here that the HTL/perovskite interface was treated as Schottky diode due to the highly doped nature of the NiO^34,35^ and to simplify the description of the device stack such that we can focus solely upon how changes in the ETL’s properties affect the JV parameters. As Driftfusion only considers one spatial dimension, we cannot explicitly model the different nanoparticles due to their nonuniform spatial distributions across the perovskite surface (see Figure S2). However, by considering how their presence would alter the rates of extraction and recombination at the perovskite/PC_61_BM interface, we can describe their effects by changing only two of the model’s parameters: the perovskite’s mobility and the surface recombination velocity of electrons at the perovskite/PC_61_BM interface.

To understand why this is the case, we refer to Figure 4. Under short circuit conditions (Figure 4a), the Al_2_O_3_ nanoparticles prevent the transfer of electrons from the perovskite to the PC_61_BM due to their insulating nature. Thus, to be extracted, electrons must diffuse around the Al_2_O_3_ nanoparticles until they reach a region where the perovskite and PC_61_BM are in direct contact.^21^ The same is not true when SnO_2_ nanoparticles are added since SnO_2_ is electronically conductive. Consequently, electrons in the devices containing the Al_2_O_3_ nanoparticles will spend longer in the region of the perovskite near the perovskite/PC_61_BM interface than they would in the devices with the SnO_2_ nanoparticles. We can include this effect in our simulations by lowering the mobility of electronic carriers in the region of the perovskite close to the perovskite/PC_61_BM interface. We stress that this lower mobility near the perovskite/ETL interface should be understood as an effective mobility, which decreases due to the fact electronic carriers must move laterally as well as in the direction parallel to the field to be extracted. As the effective mobility in this region decreases, electrons accumulate in the perovskite bulk and these accumulated electrons undergo trap assisted recombination. This results in a lower *J*_SC_, as is evident from the simulated JV curves shown in Figure 4c and matches closely the experimental data.

**Figure 4:**
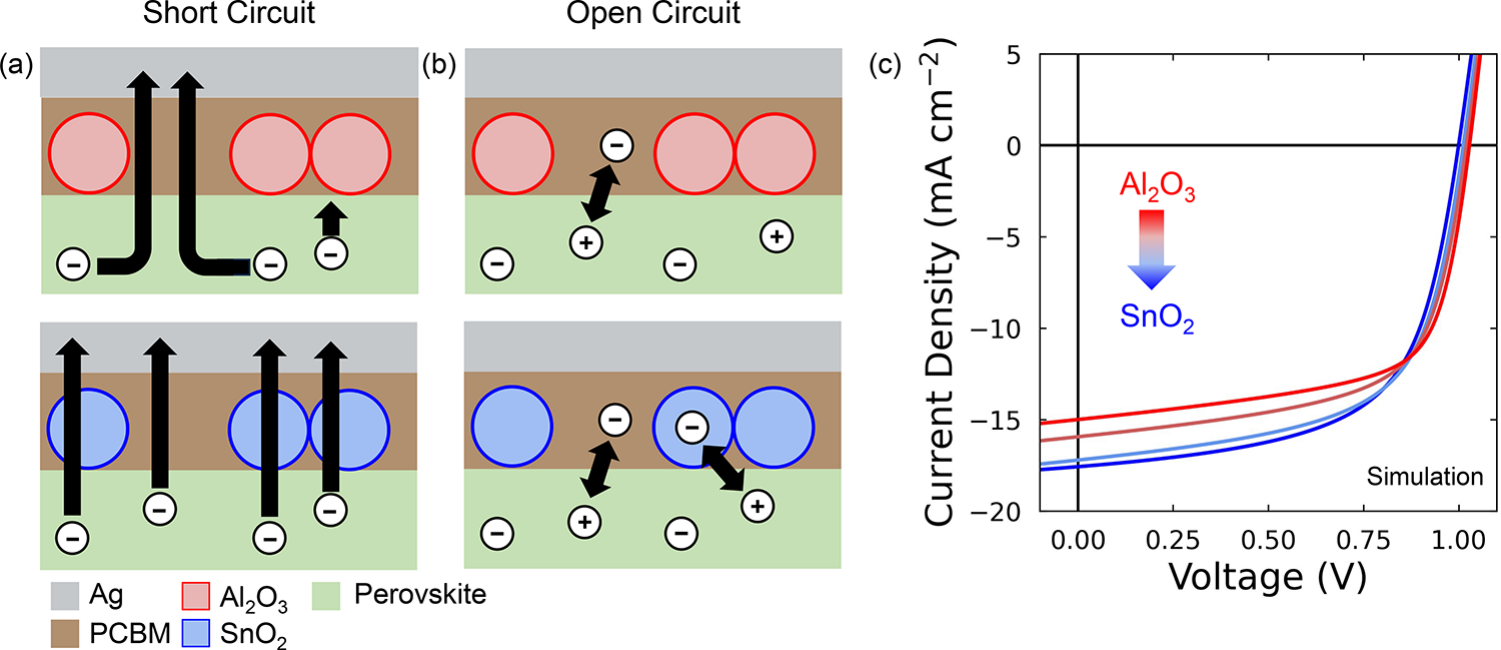
Results of Driftfusion calculations on the devices under study. Schematic diagrams of electron extraction processes at (a) *J*_SC_ and (b) *V*_OC_ of Al_2_O_3_ (top) and SnO_2_ (bottom) nanoparticle-modified devices; (c) simulated JV curves illustrating four scenarios: reduced mobility near the ETL interface and lower electron recombination current at the interface (Al_2_O_3_ case, red), increased mobility and higher recombination current at the interface (SnO_2_ case, blue), and two intermediate conditions between these two cases.

Considering now open circuit conditions (Figure 4b), here we propose that the presence of the electronically insulating Al_2_O_3_ nanoparticles reduces the fraction of the perovskite/PC_61_BM interface across which there can be interfacial recombination (i.e., recombination of a perovskite hole with an ETL electron) when compared to the case of the SnO_2_ nanoparticles.^21^ This difference can be included in our simulations by lowering the surface recombination velocity of electrons at the perovskite/PC_61_BM interface. As shown in Figure 4c, changing this parameter recreates a shift in *V*_OC_ comparable to that observed experimentally.

To further explore the impact of the different nanoparticles we performed surface photovoltage (SPV) measurements using a Kelvin probe and white light source. We measured the SPV of partial device stacks consisting of PET/ITO/HTL/Perovskite/PC_61_BM, with and without nanoparticle interface modification. The results of these measurements (Figure 5) show distinct differences in the turn-on and turn-off responses of the different samples. The positive SPV response under illumination indicates the accumulation of an excess of electrons at the surface of the samples, as expected for this sample architecture.^36^ The turn off response of all samples is biphasic with a fast initial decay close to the time scale of the measurement resolution (0.5 s), followed by a slow decay on the 10–100 s time scale. The fast phase is attributed to the fast relaxation of photogenerated charge carriers, in this case through recombination of charges across the perovskite/PC_61_BM interface.^36^ The slow phase is related to the slow recombination of trapped charge carriers and the slow migration of ions in the perovskite layer.^36–39^

**Figure 5:**
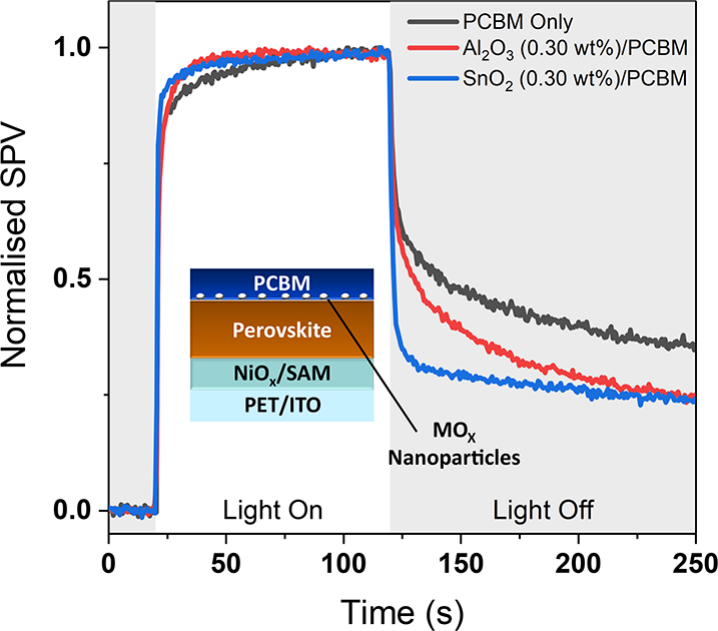
Normalized surface photovoltage responses of reference (black), Al_2_O_3_ (red), and SnO_2_ (blue) nanoparticle-modified partial device stacks under white light illumination (1/5 Sun).

The amplitude of the fast decay is much greater in the SnO_2_ modified sample, indicating more complete fast relaxation of charges. This is consistent with the SnO_2_ providing more conductive pathways for charges to recombine following the removal of the light bias. In contrast, in the Al_2_O_3_ modified sample, a greater fraction of the charge remains trapped in the PC_61_BM layer leading to a reduction in the amplitude of the fast phase. This could be a result of the insulating nature of the Al_2_O_3_ nanoparticles slowing the rate of electrons in the PC_61_BM recombining with holes in the perovskite, which is consistent with its charge blocking characteristics in device. Figure S16 gives the results of taking the first derivative of the SPV decays of the samples under study. As can be seen all samples show steep decays in the short time scales but equilibrate to similar slow rates of decay at longer time scales. This is consistent with the slow detrapping of charges and redistribution of ions in the perovskite layer discussed in previous work occurring by the same mechanisms in all samples.^36,40^

### Demonstration of Minimodules

Finally, to demonstrate the utility of this approach to large scale manufacturing we fabricated minimodules incorporating an SnO_2_ nanoparticle interlayer (Figure 6). In this scenario we found a much higher nanoparticle concentration of 1.25 wt % to be optimum, with similar results also achieved with concentrations of 0.625 and 0.75 wt % (Figure S17). We attribute this difference to the PC_61_BM needing to be near-defect free across the whole 7.2 cm^2^ of the device for acceptable performance, rather than just 0.5 cm^2^ for the previously discussed devices. No minimodules fabricated without a nanoparticle modified interface achieved current rectifying behavior. We further assessed the stability of the encapsulated modules by subjecting them to maximum power point tracking (MPPT) testing under 1 Sun illumination in ambient air (Figure 6c). The modules exhibit a *T*_80_ of approximately 150 h (as measured from the PCE at *T* = 0 h). The degradation is driven by changes in both the FF and *J*_SC_, with the FF being primarily responsible for the ∼20% increase in PCE over the first 24 h (Figure S18). Further work is being undertaken to understand the degradation mechanism of these devices.

**Figure 6:**
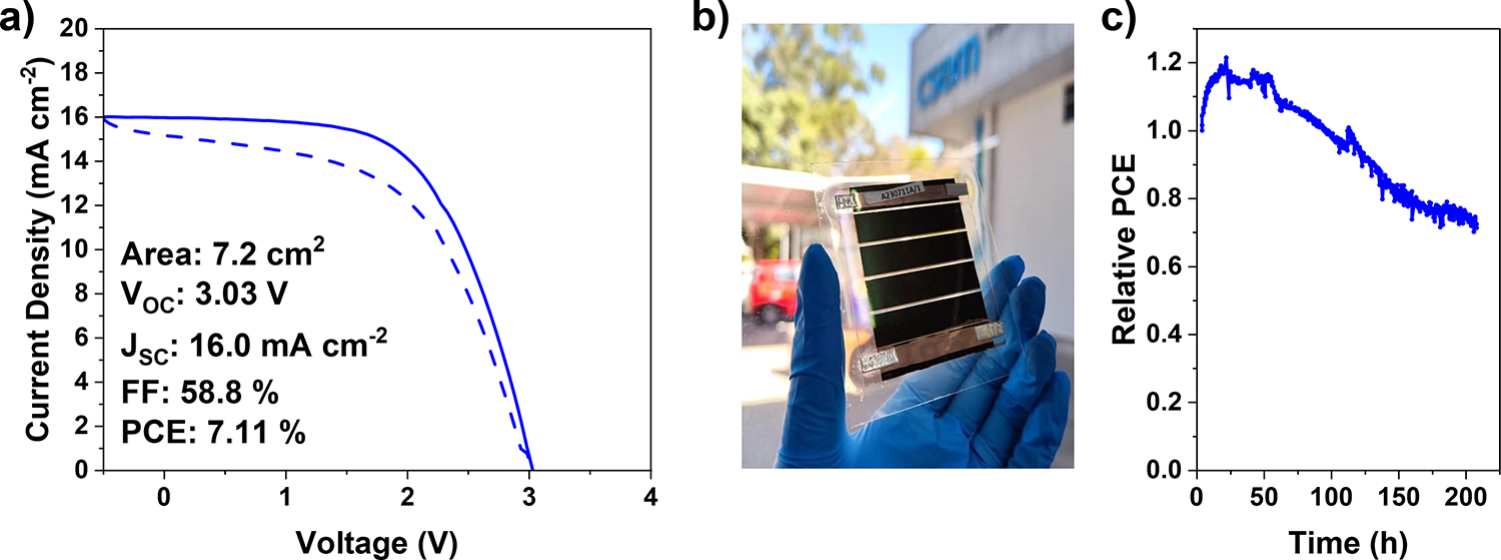
(a) JV curve of the champion minimodule of the structure PET/ITO/NiO_*x*_/MeO-2PACz/perovskite/SnO_2_(1.25 wt %)/PC_61_BM/BCP/Ag, (b) photograph of a typical encapsulated minimodule, and (c) stability of an encapsulated minimodule in air under 1 Sun illumination and MPPT conditions.

## Conclusions

In summary we have demonstrated that noncontinuous interlayers of metal oxide nanoparticles can act as an interfacial modifier in large area perovskite solar cells enabling printing of continuous and high quality PC_61_BM ETLs. Addition of the nanoparticle interlayers prevents micron scale pinhole formation in the PC_61_BM layer, significantly improving the performance and reproducibility of the perovskite solar cells. We further demonstrate the impact of the choice of nanoparticle, comparing insulating Al_2_O_3_ and semiconducting SnO_2_ nanoparticles. We show that Al_2_O_3_ nanoparticles passivate the perovskite/PC_61_BM interface but, despite a surface coverage of just 20%, also significantly hinder the extraction of charges to the PC_61_BM ETL resulting in a loss of current. Replacement of Al_2_O_3_ with SnO_2_ nanoparticles results in faster charge extraction to the ETL, producing a 24% increase in average *J*_SC_ and devices achieving PCEs of up to 11%. Finally, we demonstrated the utility of this approach with the fabrication of 7.2 cm^2^ minimodules utilizing SnO_2_ nanoparticle interlayers. This work highlights the importance of material selection for interfaces and presents a strategy for improving interface quality and manufacturing yield in large area printed perovskite solar cells.

## Methods

### Solutions

NiO_*x*_ nanoparticle solution with initial concentration of 2.5 wt % was diluted in a 1:10 proportion in ethanol prior to use. MeO2-PACz (TCI) was diluted in ethanol to achieve a final concentration of 0.33 mg/mL. PbI_2_ (TCI), formamidinium iodide (Sigma-Aldrich), CsI (Sigma-Aldrich) were weighted accordingly and dissolved in a DMF free, DMSO based solvent system with 4% L-α-phosphatidylcholine to achieve a Cs_0.17_FA_0.83_PbI_3_ solution. Likewise, PbBr_2_ (Sigma-Aldrich), CsBr (Sigma-Aldrich) and formamidinium bromide (Sigma-Aldrich) were diluted in the same solution to achieve a Cs_0.17_FA_0.83_PbBr_3_ composition. Both solutions were left stirring overnight at 60 °C. One hour prior to use, the solutions were mixed to achieve a final composition of a Cs_0.17_FA_0.83_Pb­(I_0.83_Br_0.17_)_3_.

Al_2_O_3_ (Sigma-Aldrich, nanoparticles, <50 nm particle size (DLS), 20 wt % in isopropanol) and SnO_2_ (Avantama, nanoparticles, 5 wt % in isopropanol) suspensions were made up immediately prior to use by dilution with anhydrous IPA to the required concentration. PC_61_BM (Nano-C, 99%) solutions were made up to 20 mg/mL in o-xylene and stirred overnight in an N_2_ environment at 80 °C. Bathocuproine (BCP) solutions were made up to 0.5 mg/mL in IPA in an N_2_ environment and stirred overnight at 40 °C.

### Device Fabrication

All wet film processes were carried out in ambient air within a clean room with controlled humidity (40–50% RH). A 10 cm wide PET-based web coated with ITO (200 nm, 18 Ω/sq) was used as the substrate for device fabrication. The depositions were carried out in a cleanroom environment with controlled humidity. NiO_*x*_ nanoparticle suspension was slot-die coated at 40 °C with a web speed of 1 m/min, an ink flow rate of 0.50 mL/min. The resulting film was thermally annealed in an in-line convection oven at 120 °C. Subsequently, the SAM layer was deposited on top of the NiO_*x*_ under the same conditions. Perovskite slot-die coating was performed at 100 °C with a web speed of 0.5 m/min and an ink flow rate of 0.30 mL/min. Nitrogen gas quenching was applied to aid solvent removal and enhance crystallization. The film was then thermally annealed at 150 °C in an in-line convection oven. The final perovskite film thickness was approximately 500 nm.

∼7 cm strips of the PET/ITO/NiO_*x*_/Me4-PACz/perovskite film were cut for evaporation of C_60_/BCP layers or blade coating of the Al_2_O_3_/SnO_2_, PC_61_BM and BCP layers. Al_2_O_3_ and SnO_2_ layers were blade coated using an optimized gap height of 400 μm, blade speed of 5 mm/s and bed temperature of 50 °C; for details of this optimization we direct the reader to the Supporting Information file. PC_61_BM and BCP films were blade coated using a gap height of 400 μm, blade speed of 10 mm/s and bed temperature of 60 °C. All blade coated layers were deposited from 70 μL of the respective solution per substrate (7 × 10 cm). For devices with C_60_ ETLs 20 nm of C_60_ and 5 nm of BCP were sequentially evaporated under ultrahigh vacuum. All devices were finished with a 100 nm Ag cathode deposited under ultrahigh vacuum, with a shadow mask defining a pixel area of 0.5 cm^2^ each.

Devices were encapsulated in ambient air conditions using a flexible barrier film (WVTR 10^–4^ g/m^2^ day) and a UV-curable epoxy-based adhesive in a roll-to-roll machine, reaching a final adhesive thickness of 30 μm. The encapsulation process is discussed in greater detail by Soares et al.^41^

For the fabrication of mini-modules, a web containing a mechanically defined P1 pattern was used as the substrate. All layers were deposited according to the methods set out for the smaller cells, apart from where specified in the main text. After the deposition of BCP, a 532 nm laser with a fluence of 145 mJ/cm^2^ was employed to selectively remove the layers and expose the bare ITO surface, thereby creating the P2 cut. Following the deposition of the silver cathode, the same laser was used at a reduced fluence of 29 mJ/cm^2^ to remove the metal layer, thus creating the P3 pattern.

### Characterization of PSCs

Current density–voltage (JV) curves were collected using a Keithley 2400 with the device exposed to 1 Sun illumination (Wacom WXS-156S-10 Solar Simulator) under flowing N_2_. A scan speed of 0.5 V/s was used, with the devices being first being scanned in the reverse direction from rest before being allowed to return to rest and scanned in the forward direction. To avoid previously reported light-soaking effects,^34^ devices were first soaked for 2 min under open circuit conditions before testing was carried out. Series resistances were calculated from the gradient of the device JV curve under 1 Sun around *V*_OC_ and the shunt resistances were calculated from the gradient of the device JV curve in the dark around 0 V.

Stability measurements were carried out using a custom LED solar simulator with an intensity of 1000 W m^–2^ and approximate AM1.5G spectral match. Encapsulated devices were tested in air and held under MPP conditions for the duration of the test.

External quantum efficiency measurements were carried out using a Sciencetech PTS-2-QE/ICPE.

### Scanning Electron Microscopy

Samples were mounted onto SEM stubs using carbon tape, and the edges were painted with silver paint to improve electrical contact with the stub. Images were taken on a Zeiss Gemini Sigma 300 FEG SEM instrument operating at 5 kV, with a working distance ≈5.5 mm. Image analysis to quantify the surface coverage of the nanoparticles and PC_61_BM was performed using ImageJ.^42^

### Raman Spectroscopy

Raman spectral maps were collected using a Renishaw inVia microscope in backscattering mode with the samples exposed to a flowing N_2_ atmosphere. An Ar ion laser (wavelength: 457 nm) was used to excite the samples, with exposure times and laser power optimized to increase the signal-to-noise ratio. An in-built 100 nm resolution encoded stage was used to position the sample automatically to produce the spatial maps. No degradation of the samples was observed following exposure to the laser. Spectrometer calibration was performed using an Si reference sample.

### Atomic Force Microscopy

AFM images were collected using a Park NX10 AFM in noncontact mode with Park silicon PPP-NCHR tips. The AFM was controlled using SmartScan software and the images analyzed using Gwyddion.

### Confocal PL Microscopy

PL imaging of the films was performed using an inverted fluorescence microscope (IX71, Olympus) with air objective lens (UMPlanFl 100×/0.95, Olympus) and appropriate optical filters. The samples were excited at 420 nm using a supercontinuum laser (Fianium WhiteLase, with power of 3.22 W/cm^2^), and the signal was detected with an electron-multiplying (EM) charge-coupled device (CCD) camera (iXon, Andor Technology). For the spectral measurements, the PL signal was further dispersed by using an imaging spectrograph (CLP-50LD, Bunkou Keiki) placed before the EM-CCD camera.

### Time-Resolved PL Spectroscopy

Transient photoluminescence (TRPL) measurements were collected using an Edinburgh Instruments FLS1000 fitted with a 405 nm excitation laser (6 mW cm^–2^ at 2 MHz). A long pass filter (>455 nm) was fitted between the sample and the detector to reduce the impact of reflected excitation light.

### Simulations

To perform the drift-diffusion simulations shown in Figure 4, we used Driftfusion.^33^ This software is designed to model the behavior of ordered semiconductor devices which contain up to two species of mobile ionic charge. Details of the treatment of the mobile ionic charge can be found in our previous works.^43,44^ To simulate the JV measurements, the devices were first allowed to stabilize under AM1.5G illumination at a forward bias of 1.1 V. Then the voltage was swept from 1.1 to −0.1 V at a scan rate of 10 mV s^–1^.

## Supplementary Material



## Notes

Detailed device performance statistics, SEM images, Raman mapping data, AFM images, detailed device stability data, EQE spectra, photoluminescence spectra, and details of drift-diffusion simulations (PDF)

## △

Department of Chemistry, University of Oxford, Oxford OX1 3QZ, U.K

## References

[1] Chen H., Liu C., Xu J., Maxwell A., Zhou W., Yang Y., Zhou Q., Bati A. S. R., Wan H., Wang Z., Zeng L., Wang J., Serles P., Liu Y., Teale S., Liu Y., Saidaminov M. I., Li M., Rolston N., Hoogland S., Filleter T., Kanatzidis M. G., Chen B., Ning Z., Sargent E. H. (2024). Improved Charge
Extraction in Inverted Perovskite Solar Cells with Dual-Site-Binding
Ligands. Science.

[2] Song F., Zheng D., Feng J., Liu J., Ye T., Li Z., Wang K., Liu S. F., Yang D. (2024). Mechanical Durability
and Flexibility in Perovskite Photovoltaics: Advancements and Applications. Adv. Mater..

[3] Jung H. S., Han G. S., Park N.-G., Ko M. J. (2019). Flexible Perovskite
Solar Cells. Joule.

[4] Park N. G., Zhu K. (2020). Scalable Fabrication
and Coating Methods for Perovskite Solar Cells
and Solar Modules. Nat. Rev. Mater..

[5] Weerasinghe H. C., Macadam N., Kim J.-E., Sutherland L. J., Angmo D., Ng L. W. T., Scully A. D., Glenn F., Chantler R., Chang N. L., Dehghanimadvar M., Shi L., Ho-Baillie A. W. Y., Egan R., Chesman A. S. R., Gao M., Jasieniak J. J., Hasan T., Vak D. (2024). The First
Demonstration of Entirely Roll-to-Roll Fabricated Perovskite Solar
Cell Modules under Ambient Room Conditions. Nat. Commun..

[6] Wang Y., Duan C., Lv P., Ku Z., Lu J., Huang F., Cheng Y.-B. (2021). Printing
Strategies for Scaling-up
Perovskite Solar Cells. Natl. Sci. Rev..

[7] Uddin M. A., Rana P. J. S., Ni Z., Dai X., Yu Z., Shi Z., Jiao H., Huang J. (2022). Blading of
Conformal Electron-Transport
Layers in p–i–n Perovskite Solar Cells. Adv. Mater..

[8] Caprioglio P., Smith J. A., Oliver R. D. J., Dasgupta A., Choudhary S., Farrar M. D., Ramadan A. J., Lin Y., Christoforo M. G., Ball J. M., Diekmann J., Thiesbrummel J., Zaininger K., Shen X., Johnston M. B., Neher D., Stolterfoht M., Snaith H. J. (2023). Open-Circuit and Short-Circuit Loss
Management in Wide-Gap Perovskite p-i-n Solar Cells. Nat. Commun..

[9] Küffner J., Wahl T., Schultes M., Hanisch J., Zillner J., Ahlswede E., Powalla M. (2020). Nanoparticle Wetting
Agent for Gas
Stream-Assisted Blade-Coated Inverted Perovskite Solar Cells and Modules. ACS Appl. Mater. Interfaces.

[10] Cassella E. J., Oliver R. D. J., Thornber T., Tucker S., Goodwin R., Lidzey D. G., Ramadan A. J. (2024). Alumina
Nanoparticles Enable Optimal
Spray-Coated Perovskite Thin Film Growth on Self-Assembled Monolayers
for Efficient and Reproducible Photovoltaics. J. Mater. Chem. C.

[11] Perera W. H. K., Masteghin M. G., Shim H., Davies J. D., Ryan J. L., Hinder S. J., Yun J. S., Zhang W., Jayawardena K. D. G. I., Silva S. R. P. (2023). Modification of Hydrophobic Self-Assembled Monolayers
with Nanoparticles for Improved Wettability and Enhanced Carrier Lifetimes
Over Large Areas in Perovskite Solar Cells. Sol. RRL.

[12] Jin H., Farrar M. D., Ball J. M., Dasgupta A., Caprioglio P., Narayanan S., Oliver R. D. J., Rombach F. M., Putland B. W. J., Johnston M. B., Snaith H. J. (2023). Alumina Nanoparticle Interfacial
Buffer Layer for Low-Bandgap Lead-Tin Perovskite Solar Cells. Adv. Funct. Mater..

[13] Li J., Meng X., Huang Z., Dai R., Sheng W., Gong C., Tan L., Chen Y. (2022). A Regularity-Based
Fullerene Interfacial Layer for Efficient and Stable Perovskite Solar
Cells via Blade-Coating. Adv. Funct. Mater..

[14] Soares, G. de A. ; Marques, A. dos S. ; Guimarães, F. A. ; Bicalho, I. S. ; Fernandes, S. L. ; Alves, G. X. G. ; Pereira, D. C. ; Miranda, B. H. de S. ; Vilela, M. L. P. ; Rodrigues, J. F. ; Silva, T. M. G. da ; Martins, J. L. da S. ; Queiroz, R. V. de ; Bagnis, D. Construção de um painel fotovoltaico flexível de perovskita. Anais Congresso Brasileiro De Energia Solar - CBENS, 2024. 10.59627/cbens.2024.2388.

[15] International Labour Organisation. ICSC 0084: o-Xylene. https://chemicalsafety.ilo.org/dyn/icsc/showcard.display?p_card_id=0084 (accessed 2026–01–21).

[16] International Labour Organisation. ICSC 0642: Chlorobenzene. https://chemicalsafety.ilo.org/dyn/icsc/showcard.display?p_lang=en&p_card_id=0642&p_version=2 (accessed 2026–01–21).

[17] Chung K. T., Reisner J. H., Campbell E. R. (1983). Charging Phenomena in the Scanning
Electron Microscopy of Conductor-Insulator Composites: A Tool for
Composite Structural Analysis. J. Appl. Phys..

[18] Dzwilewski A., Wågberg T., Edman L. (2009). Photo-Induced and Resist-Free Imprint
Patterning of Fullerene Materials for Use in Functional Electronics. J. Am. Chem. Soc..

[19] Li Z., Wong H. C., Huang Z., Zhong H., Tan C. H., Tsoi W. C., Kim J. S., Durrant J. R., Cabral J. T. (2013). Performance
Enhancement of Fullerene-Based Solar Cells by Light Processing. Nat. Commun..

[20] Lee M. M., Teuscher J., Miyasaka T., Murakami T. N., Snaith H. J. (2012). Efficient
Hybrid Solar Cells Based on Meso-Superstructured Organometal Halide
Perovskites. Science.

[21] Peng W., Mao K., Cai F., Meng H., Zhu Z., Li T., Yuan S., Xu Z., Feng X., Xu J., McGehee M. D., Xu J. (2023). Reducing Nonradiative
Recombination
in Perovskite Solar Cells with a Porous Insulator Contact. Science.

[22] Golubev T., Liu D., Lunt R., Duxbury P. (2019). Understanding the Impact of C60 at
the Interface of Perovskite Solar Cells via Drift-Diffusion Modeling. AIP Adv..

[23] Glück N., Hill N. S., Giza M., Hutter E., Grill I., Schlipf J., Bach U., Müller-Buschbaum P., Hartschuh A., Bein T., Savenije T., Docampo P. (2024). The Balancing
Act between High Electronic and Low Ionic Transport Influenced by
Perovskite Grain Boundaries. J. Mater. Chem.
A.

[24] Tress W., Petrich A., Hummert M., Hein M., Leo K., Riede M. (2011). Imbalanced Mobilities Causing S-Shaped IV Curves in Planar Heterojunction
Organic Solar Cells. Appl. Phys. Lett..

[25] Min H., Lee D. Y., Kim J., Kim G., Lee K. S., Kim J., Paik M. J., Kim Y. K., Kim K. S., Kim M. G., Shin T. J., Il Seok S. (2021). Perovskite
Solar Cells with Atomically
Coherent Interlayers on SnO2 Electrodes. Nature.

[26] Almora O., Cabrera C. I., Garcia-Cerrillo J., Kirchartz T., Rau U., Brabec C. J. (2021). Quantifying the Absorption Onset in the Quantum Efficiency
of Emerging Photovoltaic Devices. Adv. Energy
Mater..

[27] Ramadan A. J., Oliver R. D. J., Johnston M. B., Snaith H. J. (2023). Methylammonium-Free
Wide-Bandgap Metal Halide Perovskites for Tandem Photovoltaics. Nat. Rev. Mater..

[28] Du T., Xu W., Xu S., Ratnasingham S. R., Lin C.-T., Kim J., Briscoe J., McLachlan M. A., Durrant J. R. (2020). Light-Intensity
and Thickness Dependent Efficiency of Planar Perovskite Solar Cells:
Charge Recombination versus Extraction. J. Mater.
Chem. C.

[29] Min L., Zhou Y., Sun H., Guo L., Wang M., Cao F., Tian W., Li L. (2024). Carrier Dynamic Identification Enables
Wavelength and Intensity Sensitivity in Perovskite Photodetectors. Light Sci. Appl..

[30] Xu W., Du T., Sachs M., Macdonald T. J., Min G., Mohan L., Stewart K., Lin C. T., Wu J., Pacalaj R., Haque S. A., McLachlan M. A., Durrant J. R. (2022). Asymmetric Charge
Carrier Transfer and Transport in Planar Lead Halide Perovskite Solar
Cells. Cell Reports Phys. Sci..

[31] Péan E. V., Dimitrov S., De Castro C. S., Davies M. L. (2020). Interpreting Time-Resolved
Photoluminescence of Perovskite Materials. Phys.
Chem. Chem. Phys..

[32] Chen X., Kamat P. V., Janáky C., Samu G. F. (2024). Charge Transfer
Kinetics in Halide Perovskites: On the Constraints of Time-Resolved
Spectroscopy Measurements. ACS Energy Lett..

[33] Calado P., Gelmetti I., Hilton B., Azzouzi M., Nelson J., Barnes P. R. F. (2020). Driftfusion: An Open Source Code for Simulating Ordered
Semiconductor Devices with Mixed Ionic-Electronic Conducting Materials
in One-Dimension. J. Comput. Electron..

[34] Henderson C., Luke J., Bicalho I. S., Correa L., Yang E. J., Rimmele M., Demetriou H., Chin Y., Lan T., Heutz S., Gasparini N., Heeney M., Bagnis D., Kim J. (2023). Charge Transfer Complex
Formation between Organic Interlayers Drives
Light-Soaking in Large Area Perovskite Solar Cells. Energy Environ. Sci..

[35] Di
Girolamo D., Di Giacomo F., Matteocci F., Marrani A. G., Dini D., Abate A. (2020). Progress, Highlights
and Perspectives on NiO in Perovskite Photovoltaics. Chem. Sci..

[36] Daboczi M., Hamilton I., Xu S., Luke J., Limbu S., Lee J., McLachlan M. A., Lee K., Durrant J. R., Baikie I. D., Kim J.-S. (2019). Origin of Open-Circuit
Voltage Losses in Perovskite
Solar Cells Investigated by Surface Photovoltage Measurement. ACS Appl. Mater. Interfaces.

[37] Daboczi, M. Origin of Charge Carrier Recombination Losses in Perovskite-Based Solar Cells Revealed by Interfacial Energetics and Surface Photovoltage; Imperial College London, 2020.

[38] Pockett A., Eperon G. E., Sakai N., Snaith H. J., Peter L. M., Cameron P. J. (2017). Microseconds, Milliseconds and Seconds: Deconvoluting
the Dynamic Behaviour of Planar Perovskite Solar Cells. Phys. Chem. Chem. Phys..

[39] Leijtens T., Eperon G. E., Barker A. J., Grancini G., Zhang W., Ball J. M., Kandada A. R. S., Snaith H. J., Petrozza A. (2016). Carrier Trapping
and Recombination: The Role of Defect Physics in Enhancing the Open
Circuit Voltage of Metal Halide Perovskite Solar Cells. Energy Environ. Sci..

[40] Daboczi M., Ratnasingham S. R., Mohan L., Pu C., Hamilton I., Chin Y.-C., McLachlan M. A., Kim J.-S. (2021). Optimal Interfacial
Band Bending Achieved by Fine Energy Level Tuning in Mixed-Halide
Perovskite Solar Cells. ACS Energy Lett..

[41] Soares G. A., Bicalho I. S., Castro-Hermosa S., Corrêa L. de Q., Miranda B. H. S., Marques A. dos S., Fernandes S. L., Cunha T., de Freitas V. V., Vilaça R. de Q., Wouk L., Bagnis D. (2024). A Comparative Study of Acrylic and
Epoxy-Based Adhesives for Perovskite Solar Cells Encapsulation. Sol. Energy.

[42] Schneider C. A., Rasband W. S., Eliceiri K. W. (2012). NIH Image
to ImageJ: 25 Years of
Image Analysis. Nat. Methods.

[43] Xu W., Hart L. J. F., Moss B., Caprioglio P., Macdonald T. J., Furlan F., Panidi J., Oliver R. D. J., Pacalaj R. A., Heeney M., Gasparini N., Snaith H. J., Barnes P. R. F., Durrant J. R. (2023). Impact of Interface
Energetic Alignment and Mobile Ions on Charge Carrier Accumulation
and Extraction in P-i-n Perovskite Solar Cells.. Adv. Energy Mater..

[44] Hart L. J. F., Angus F. J., Li Y., Khaleed A., Calado P., Durrant J. R., Djurišić A. B., Docampo P., Barnes P. R. F. (2024). More Is Different: Mobile Ions Improve the Design Tolerances
of Perovskite Solar Cells.. Energy Environ.
Sci..

