# Supplementary material for: Nanoparticle-Mediated Interface Engineering for Uniform, Reproducible Electron Transport Layers in Scalable Perovskite Solar Cells
Source: ACS Appl Mater Interfaces. 2026 Feb 18;18(8):12738–48. doi: 10.1021/acsami.5c24295 (PMC12964338; doi:10.1021/acsami.5c24295)
Supplement: Supplementary file 1 [file am5c24295_si_001.pdf]

Supporting Information

Nanoparticle-Mediated Interface Engineering for Uniform, Reproducible Electron Transport Layers in Scalable Perovskite Solar Cells

Charlie Henderson,[1,†] Adriano S. Marques,[2] Izabela S. Bicalho,[2] Lucy J. F. Hart,[1,3] Amy Monahan,[4] Katherine Stewart,[1] Koki Asano,[6] Tianhao Lan,[1] Martin Vacha,[6] Molly M. Stevens,[4,5] Piers R. F. Barnes,[1] Diego Bagnis,[2] and Ji-Seon Kim[1,†,]*

1. Department of Physics and Centre for Processable Electronics, Imperial College London, London, SW7 2AZ, UK
2. Oninn Centro de Inovações, CEP 31035536 Belo Horizonte, MG, Brazil
3. Department of Chemistry and Centre for Processable Electronics, Imperial College London, London, W12 0BZ, UK
4. Department of Materials, Department of Bioengineering and Institute of Biomedical Engineering, Imperial College London, London, SW7 2AZ, UK
5. Kavli Institute for Nanoscience Discovery, Department of Physiology, Anatomy and Genetics, Department of Engineering Science, University of Oxford, Oxford, OX1 3QU, UK
6. Department of Materials Science and Engineering, School of Materials and Chemical Technology, Institute of Science Tokyo, Tokyo 152-8552, Japan

[†]Current address: Department of Chemistry, University of Oxford, Oxford, OX1 3QZ, UK

Corresponding Author: Professor Ji-Seon Kim, ji-seon.kim@imperial.ac.uk

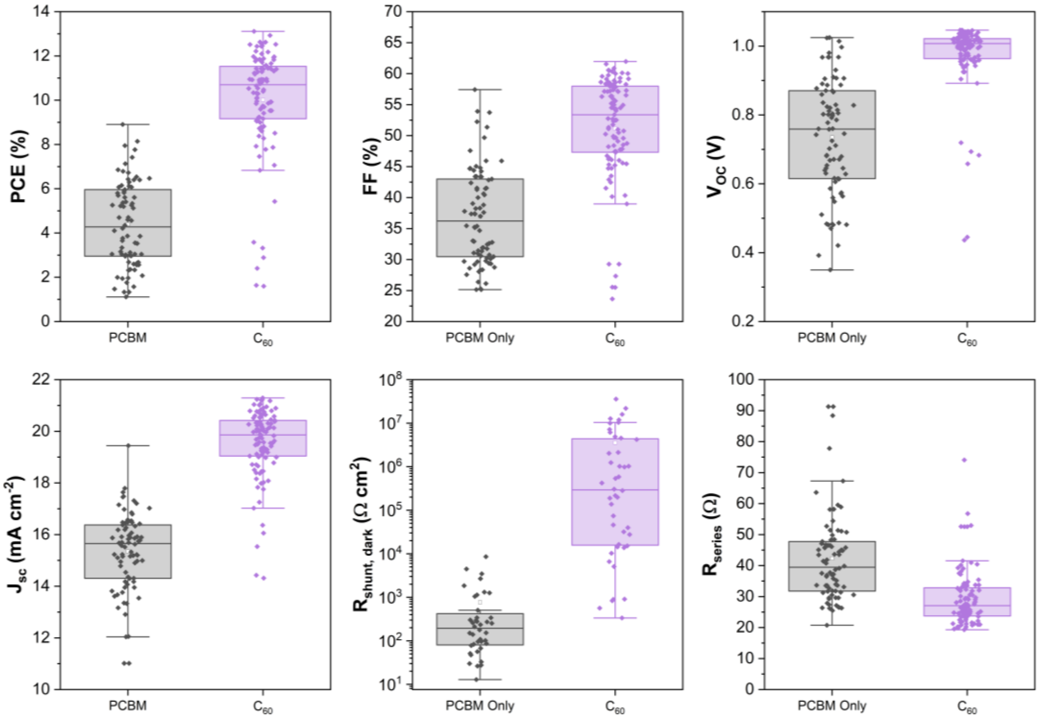

Figure S1: Device performance statistics comparing cells with $PC_{61}BM$ and C_{60} ETLs. Devices with PCE<1% have been removed. 38% of fabricated $PC_{61}BM$ based devices had PCEs<1%, compared to 4% of C_{60} based devices.

ETL Type	V_{oc}, V (Champion)	J_{sc}, mA cm^{-2} (Champion)	FF, % (Champion)	PCE, % (Champion)	PCE, % All Devices
**C_{60}**	0.97±0.10 (1.01)	19.41±1.92 (21.04)	51.2±9.1 (61.5)	9.93±2.53 (13.11)	9.56±3.12
**$PC_{61}BM$ Only**	0.73±0.17 (1.02)	15.34±1.53 (15.15)	36.9±7.7 (57.4)	4.39±1.90 (8.90)	2.93±2.53
**Al_2O_3 (0.3 wt%)/$PC_{61}BM$**	0.99±0.07 (1.02)	13.96±1.71 (17.28)	54.4±8.4 (62.2)	7.64±1.65 (10.93)	6.24±3.29
**SnO_2 (0.3 wt%)/$PC_{61}BM$**	0.98±0.10 (1.00)	17.75± 1.24 (19.07)	50.7±6.9 (57.8)	8.95±1.87 (10.99)	8.18±3.05

Table S1: Performance statistics showing the average key performance parameters of the four main device types studied in this work. Also shown is the performance parameter of the device with the highest PCE (champion) of each device type. Devices with PCE<1% were not considered for these statistics, aside from the "All Devices" column which included every device fabricated for this work.

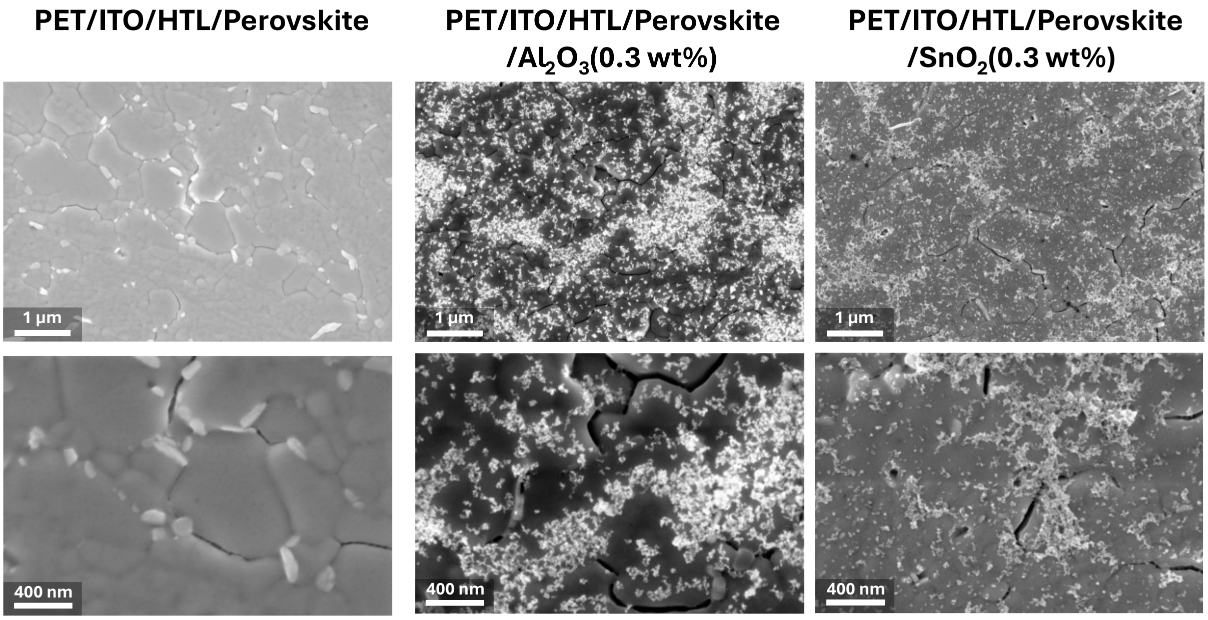

Figure S2: SEM images of R2R perovskite films without and with metal oxide nanoparticles. The nanoparticles form non continuous layers at the optimised concentrations. The increased brightness of the Al_2O_3 nanoparticles compared to the SnO_2 nanoparticles is a result of charging of the Al_2O_3 nanoparticles by incident electrons from the field emission gun.

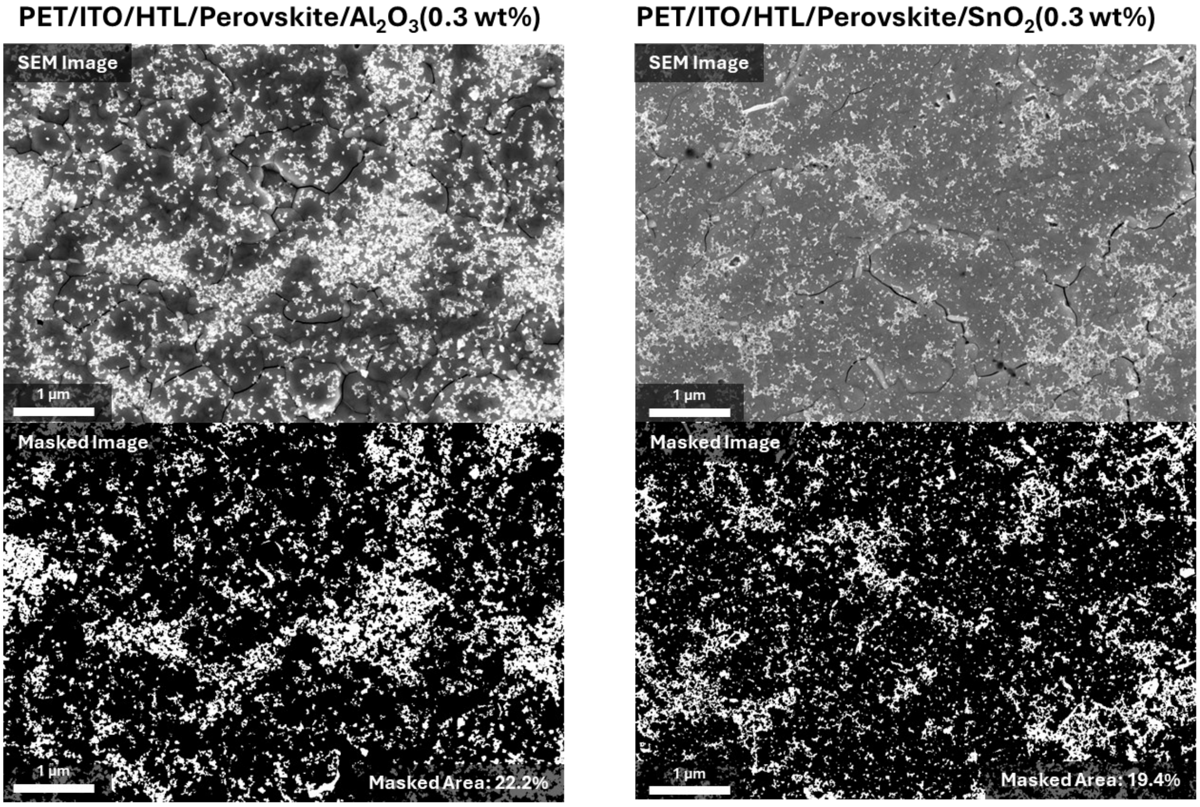

Figure S3: SEM images (top) and masked images (bottom) of nanoparticle modified perovskite films. The masked images show only the areas covered by nanoparticles

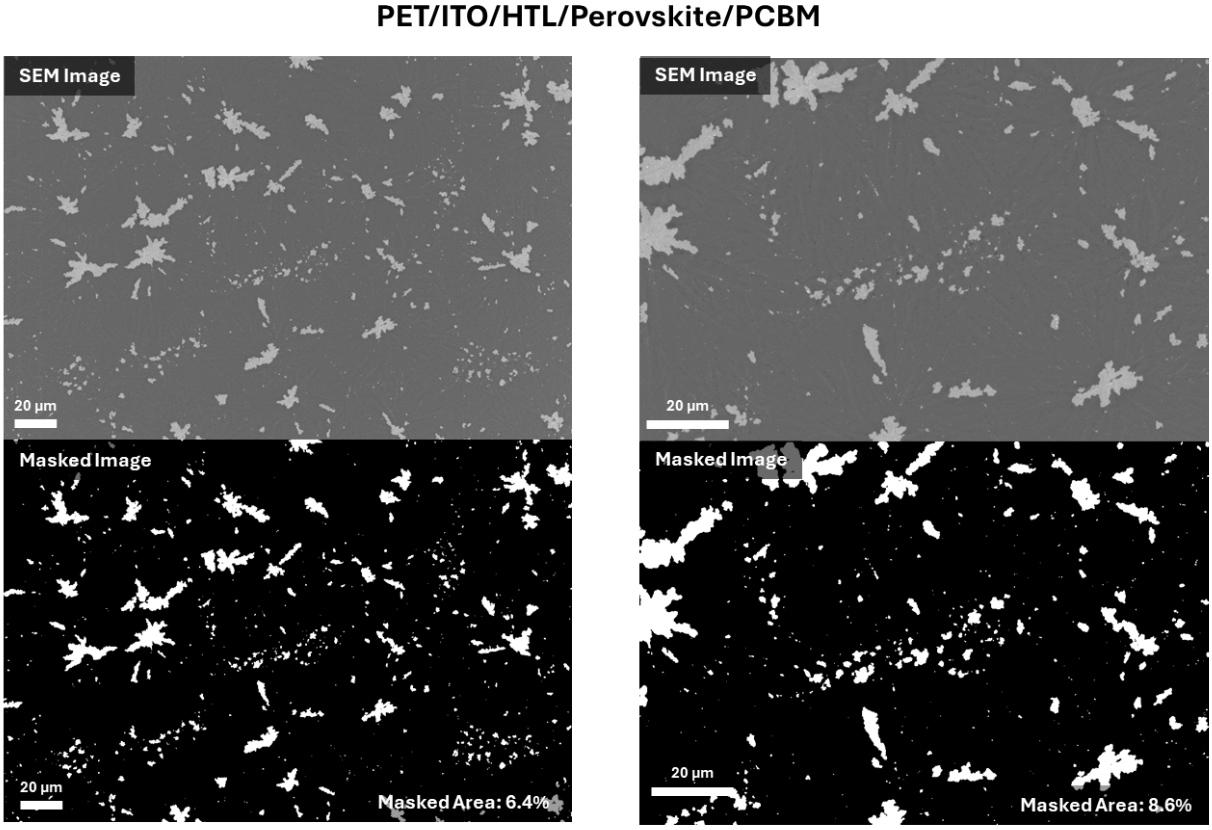

Figure S4: SEM images (top) and masked images (bottom) two areas of a perovskite/$PC_{61}BM$ film. The masked images show the areas not covered by the $PC_{61}BM$.

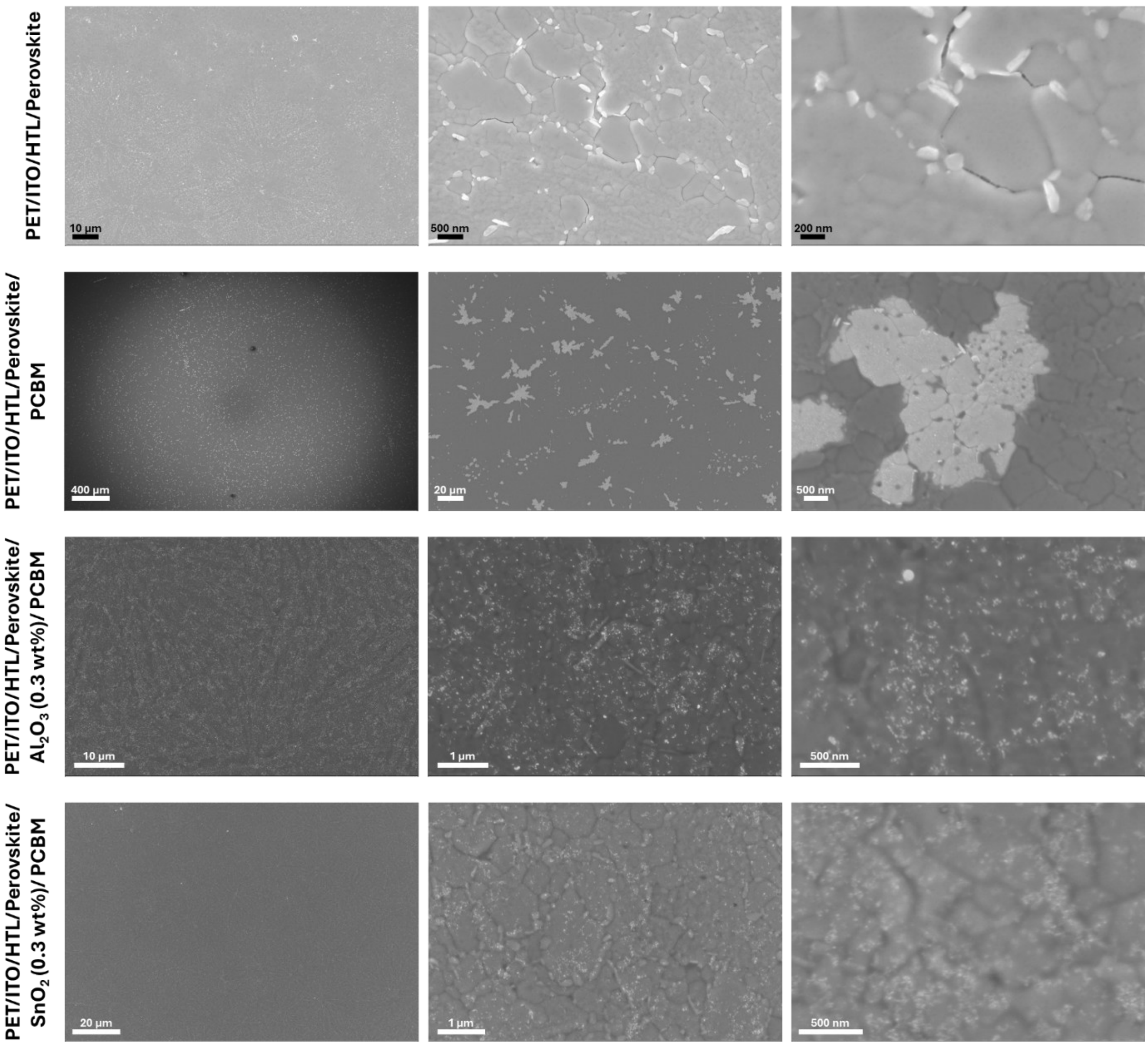

Figure S5: SEM images of, from top to bottom, perovskite only, perovskite/$PC_{61}BM$, perovskite/Al_2O_3 (0.3 wt%)/$PC_{61}BM$ and perovskite/Sno_2 (0.3 wt%)/$PC_{61}BM$ films. The images are at different length scales with the left column showing larger areas of the films with increasing magnification in the centre then righthand columns. Inhomogeneity in the perovskite/$PC_{61}BM$ films extends across the whole film, as can be seen in the ~10 mm^2 image in the first column of the second row.

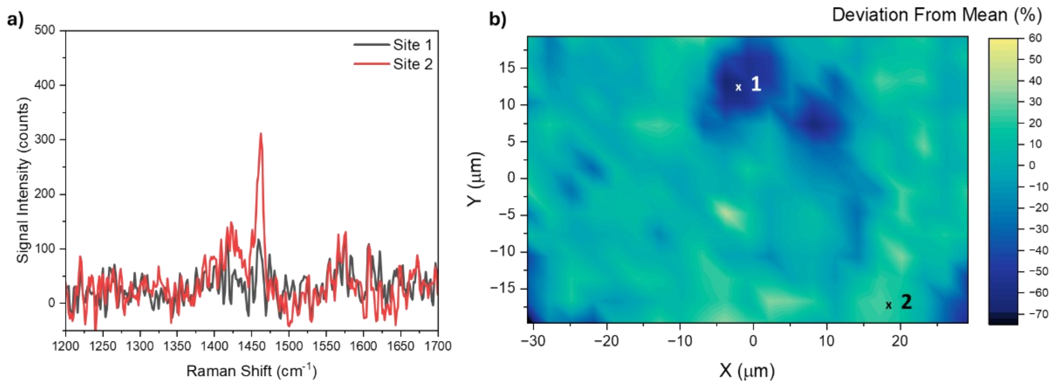

Figure S6: a) Raman spectra taken from two points (Site 1 and Site 2) on a perovskite/$PC_{61}BM$ only film; b) Raman intensity map of a perovskite/$PC_{61}BM$ film showing the locations of Site 1 and Site 2.

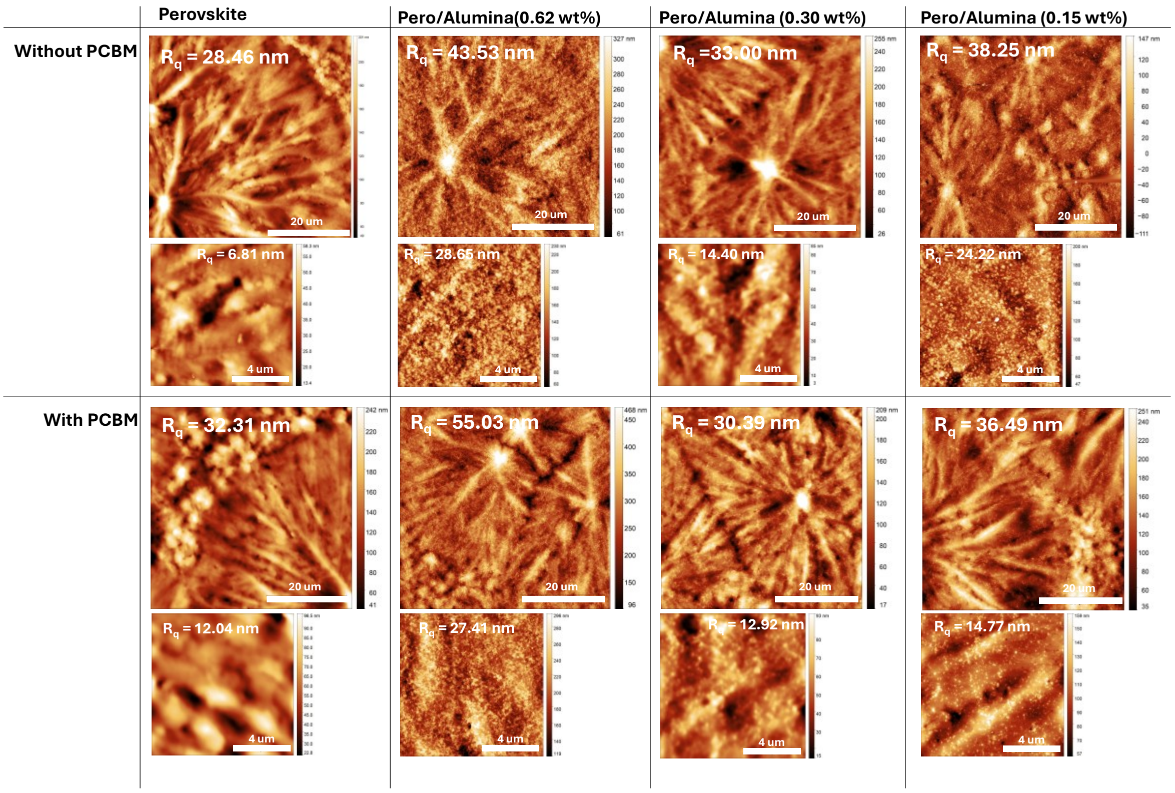

Figure S7: AFM images of perovskite films without (top) and with (bottom) $PC_{61}BM$ with different surface coverages of alumina nanoparticles.

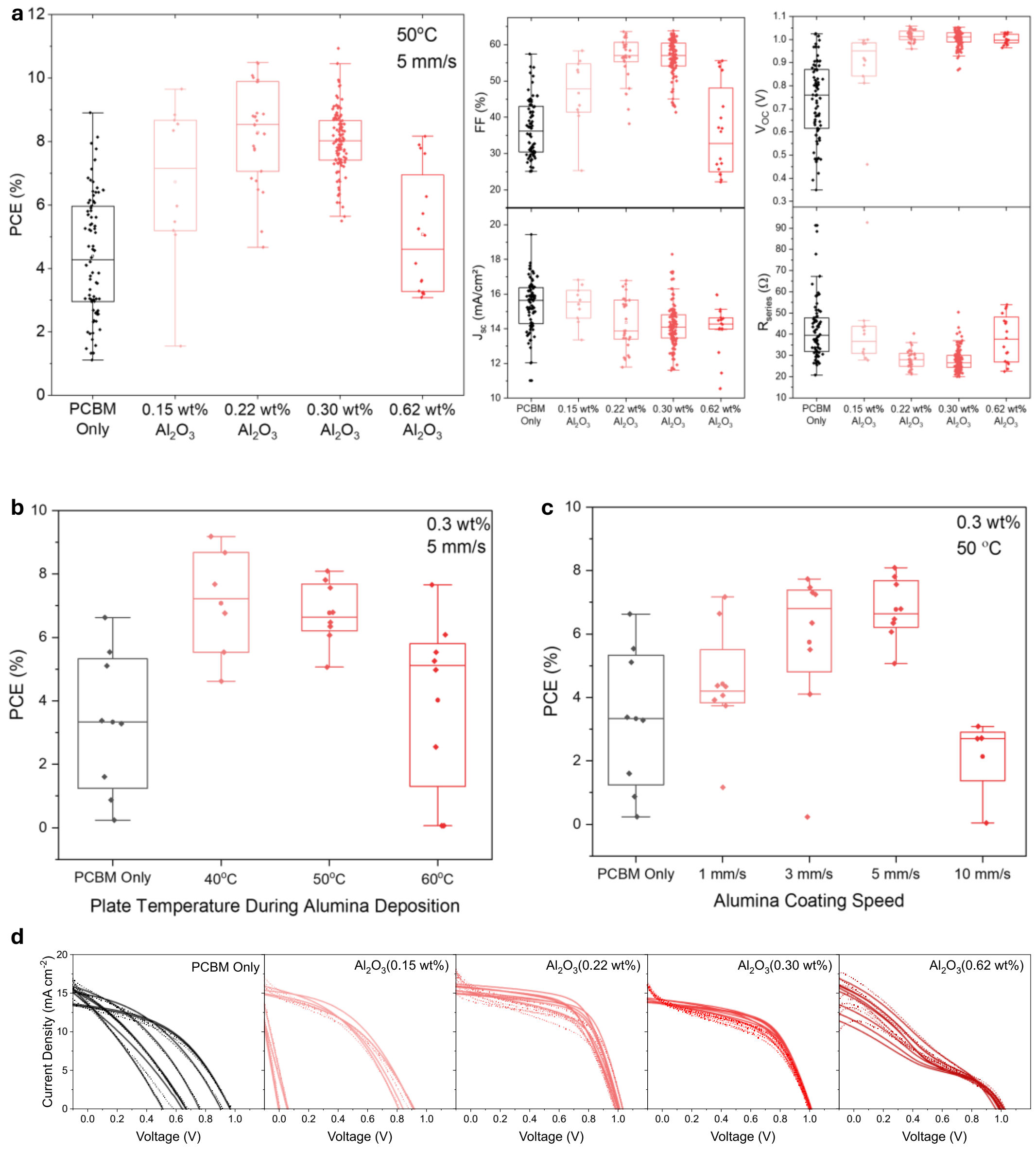

Figure S8: Optimisation of the alumina nanoparticle modified devices. a) Detailed results of concentration optimisation; b) results of blade coater plate temperature optimisation; c) results of coating blade speed optimisation; d) representative JV curves from each Al_2O_3 nanoparticle interlayer concentration.

Discussion of Calculated R_{series}

Devices with low concentrations of Al_2O_3 nanoparticles have lower calculated R_{series} values than the reference. This result is potentially unreliable as R_{series} was calculated from the gradient of the JV curve around V_{OC}, and therefore the increased R_{series} calculated for the reference devices is likely a result of low R_{shunt} and FF, meaning there is still significant slope in the JV curve at open circuit. In the 0.62 wt% Al_2O_3 nanoparticle devices R_{series} is increased due to high surface coverage of the perovskite surface resulting in poor extraction.

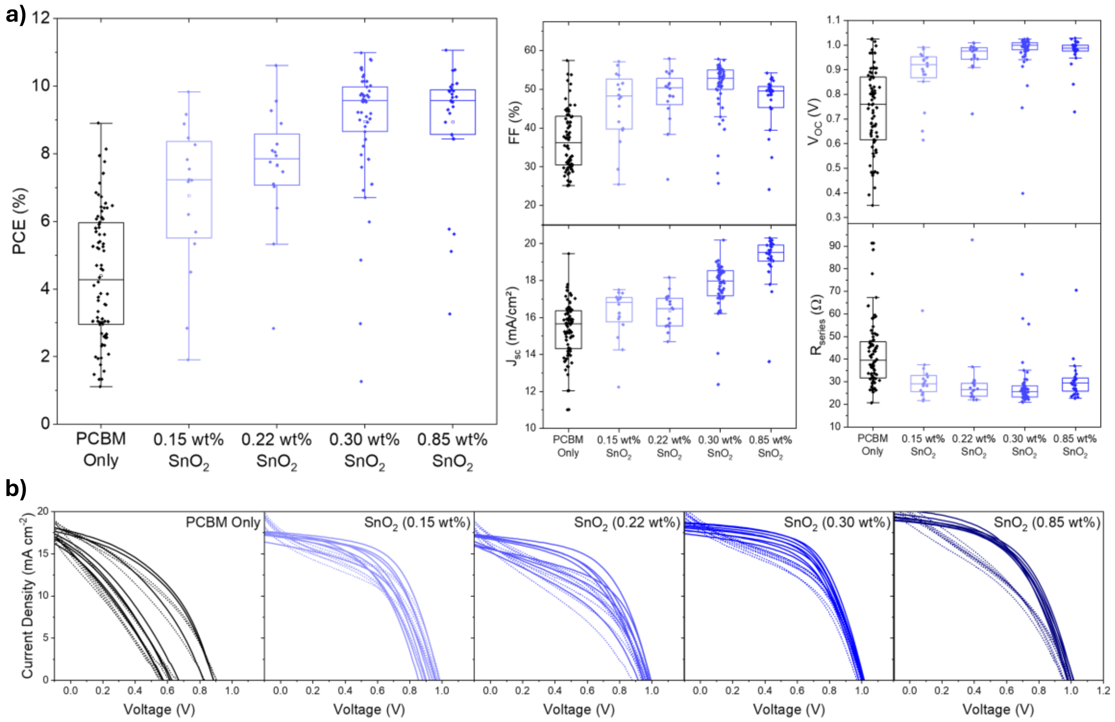

Figure S9: Results of concentration optimisation of the SnO_2 nanoparticle modified devices. a) Statistics of key solar cell performance parameters, b) representative JV curves.

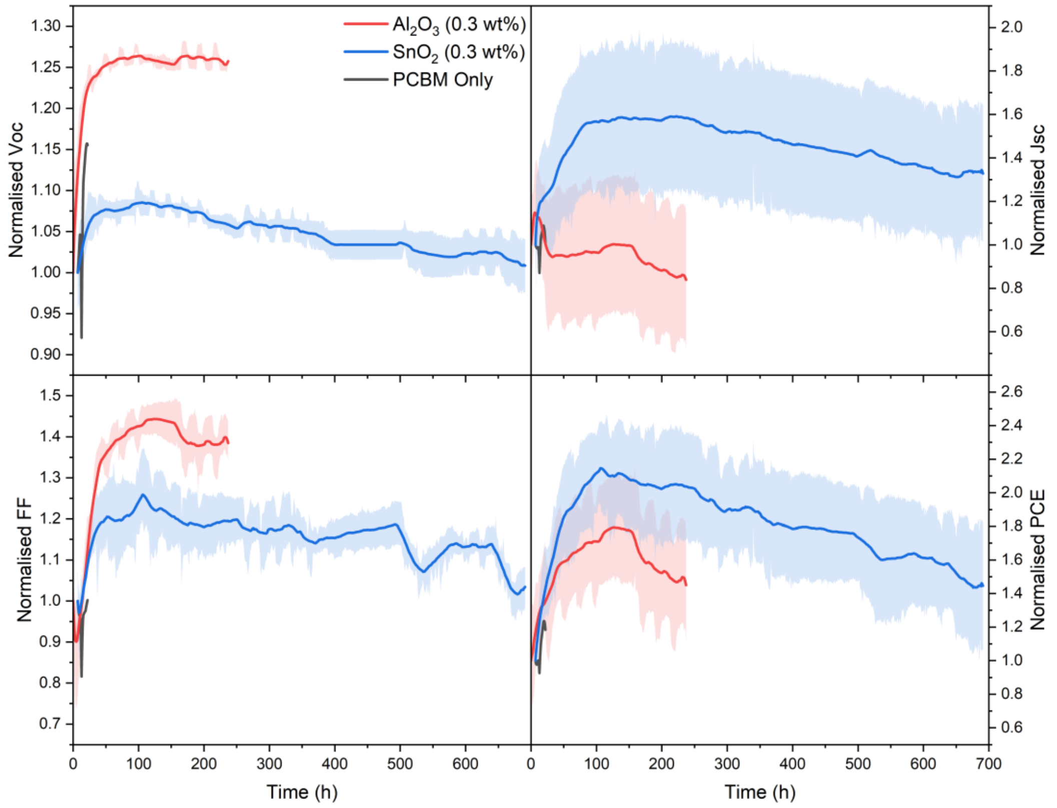

Figure S10: Results of maximum power point tracking (MPPT) stability testing of encapsulated devices under 1 Sun conditions in air. The solid lines represent the mean values of devices and the shaded area represents the range. It should be noted that the very short measurement time for the reference device is due to rapid intrinsic degradation of the device, with the PCE dropping below 1% almost immediately and faster than the temporal resolution of the measurement system. The automated stability setup disconnects devices once their performance falls below this threshold, preventing the acquisition of longer-term data.

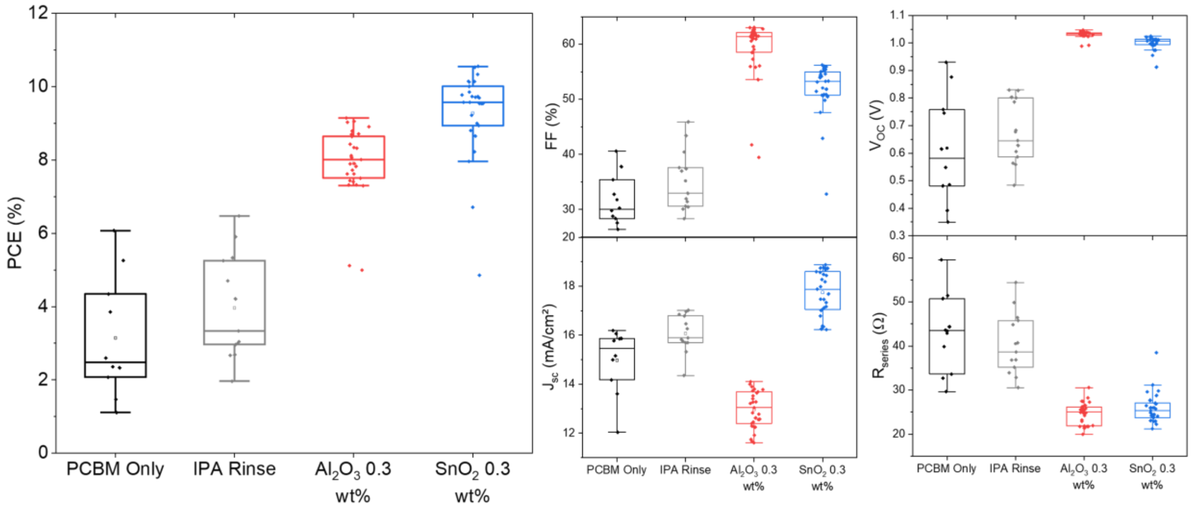

Figure S11: Comparison of optimised nanoparticle modified devices with pre-treatment of the perovskite film with IPA prior to $PC_{61}BM$ coating. Neat IPA was bladecoated using the same parameters as for the nanoparticle suspensions in IPA.

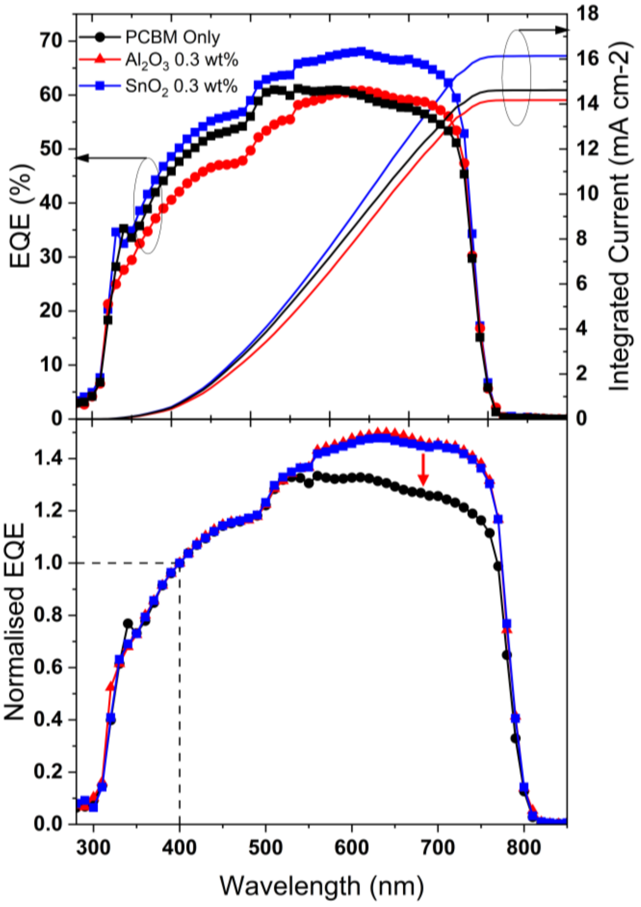

Figure S12: (Top) EQE spectra of reference (black), Al_2O_3 modified (red) and SnO_2 (blue) modified devices; also shown is the integrated current calculated from each spectrum. (Bottom) Normalised (at 400 nm) EQE spectra.

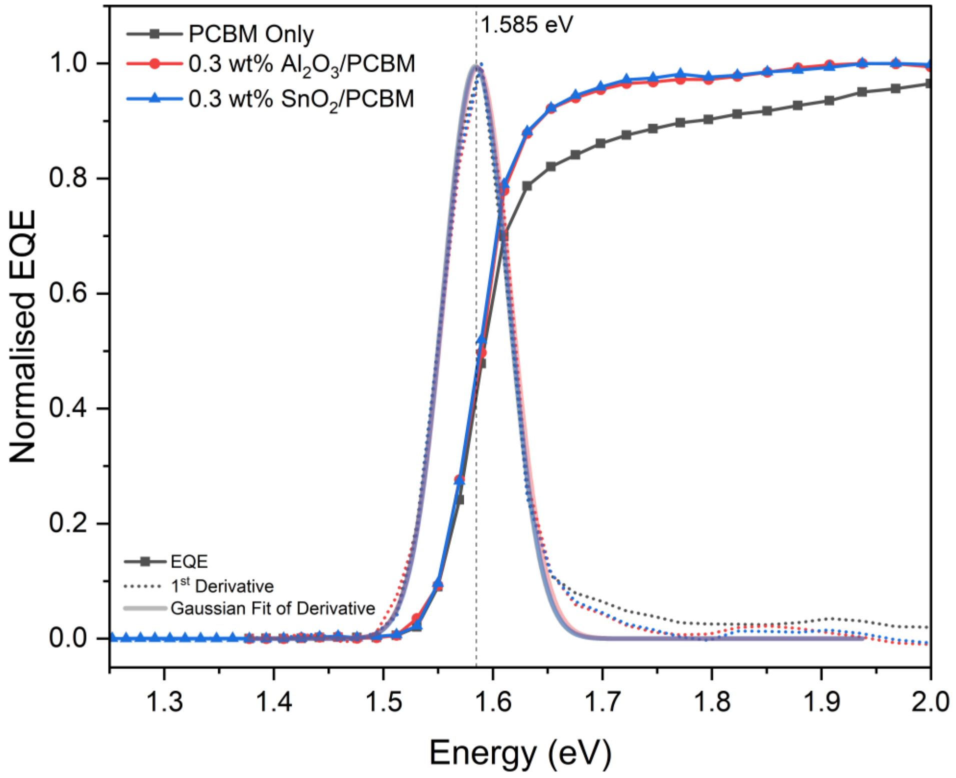

Figure S13: EQE spectra and their first derivatives around the onset energy. The photovoltaic band gap of the perovskite device was calculated from the peak of a gaussian fit of the first derivative.[1,2]

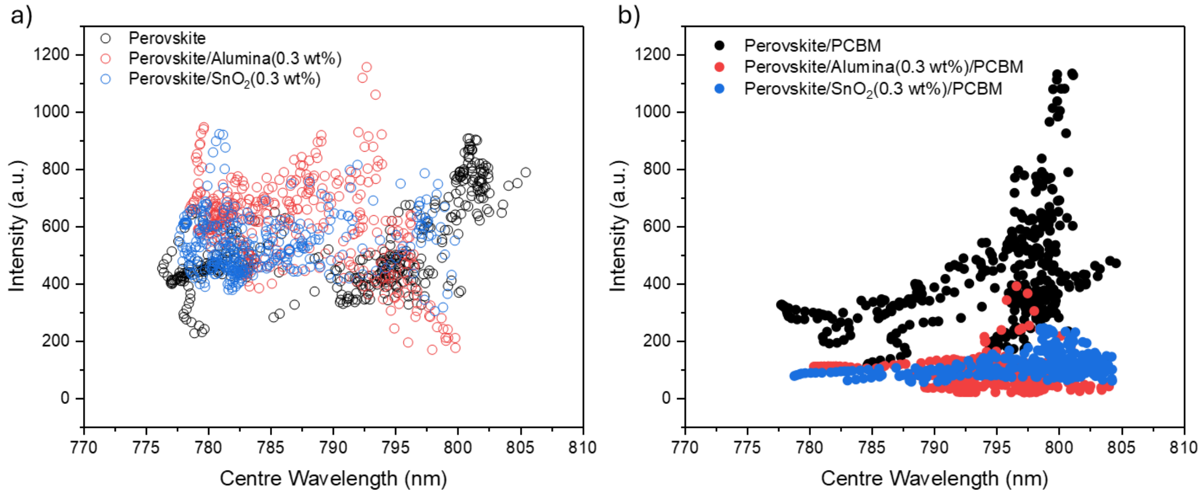

Figure S14: Results of confocal photoluminescence mapping. Shown are plots of PL peak intensity versus peak centre wavelength for 363 points across a 25x25 μm area of a) reference and nanoparticle modified perovskite films and b) reference and nanoparticle modified perovskite films with $PC_{61}BM$.

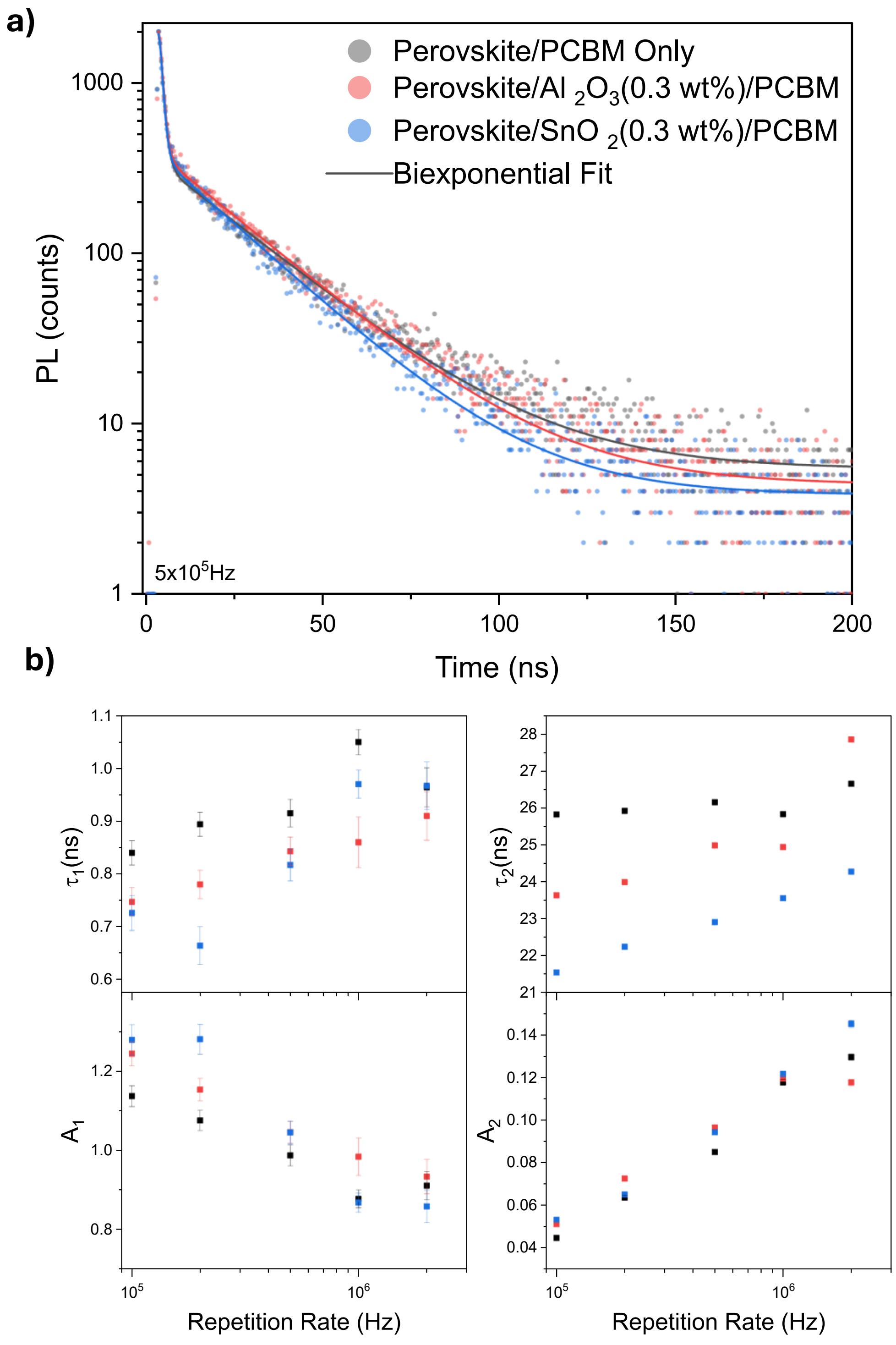

Figure S15: Transient PL (TRPL) analysis of key samples; a) TRPL decays (excitation: 405 nm) of the reference sample and optimised nanoparticle modified samples. Spectra were collected on partial device stacks (structure: PET/ITO/HTL/Perovskite/Nanoparticles/PCBM), with illumination from the top side and a laser repetition rate of $5x10^5$ Hz; b) results of fitting TRPL decays collected using a range of laser repetition rates with a simple biexponential decay model given in equation S1 where *PL* is the photoluminescence intensity, A_x is the amplitude of a decay component, and τ_x is the decay component lifetime.

$$PL(t) = A_1 e^{-t/\tau_1} + A_2 e^{-t/\tau_2} \qquad \text{(Equation S1)}$$

Parameter	Value	Ref
Perovskite Bandgap	1.60 eV	[3]
Perovskite Valance Band Energy	-5.4 eV	[3]
Perovskite Thickness	500 nm	a
Perovskite Carrier Mobility	1 $cm^2 V^{-1} s^{-1}$	[4]
Shockley-Read-Hall Lifetime	200 ns	-
Radiative Recombination Rate	5 x 10^{-11} $cm^{-3} s^{-1}$	[5]
Perovskite Relative Permittivity	25	[6]
Perovskite Effective Density of States	5 x 10^{18} cm^{-3}	[7]
Perovskite Mobile Ion Density	5 x 10^{17} cm^{-3}	-
ETL Bandgap	2.00 eV	[8]
ETL Conduction Band / LUMO Energy	-4.0 eV	b
ETL Fermi Level	-5.0 eV	c
ETL Thickness	30 nm	d
ETL Carrier Mobility	5 x 10^{-4} $cm^2 V^{-1} s^{-1}$	[9]
ETL Relative Permittivity	4.5	e
ETL Effective Density of States	1 x 10^{20} cm^{-3}	[10]
Surface Recombination Velocity of Holes at the Perovskite/ETL Interface	50 cms^{-1}	-
Cathode Work Function	-4.25 V	-
Surface Recombination Velocity of Electrons at the Perovskite/HTL Interface	50 cms^{-1}	-
Extraction Velocity of Holes at the Perovskite/HTL Interface	10^5 cms^{-1}	-
Anode Work Function	-5.25 V	-

a) Measured value.
b) Values in the range -4.2 eV to -3.9 eV have been reported for $PC_{61}BM$.[8,11] We chose the value in this range which best reproduced the observed JV curves.
c) $PC_{61}BM$ was assumed to be an intrinsic semiconductor.
d) Typical thickness for $PC_{61}BM$ ETLs.
e) Typical value for organic semiconductors.

Table S2: Parameters held constant across all the drift-diffusion simulations shown in Figure 4 of the main text. The perovskite was assumed to be intrinsic and trap states (both bulk and interfacial) were assumed to lie midgap. Where no comment or reference is given, parameters were tuned to qualitatively reproduce the behaviour observed in the JV measurements. Due to highly doped and selective nature of the NiO/SAM HTL and to focus our simulations on the effects of changing properties of the ETL, we

have treated the perovskite/HTL interface as a Schottky diode (i.e., perovskite metal interface where selectivity has been ensured by tuning the surface recombination velocities).

Simulation	Electronic Carrier Mobility ($cm^2 V^{-1} s^{-1}$)	Surface Recombination Velocity of Electrons at the Perovskite/ETL Interface ($cm s^{-1}$)
**1 (More SnO_2 like)**	1.0	10.0
**2**	0.35	8.5
**3**	0.15	5.0
**4 (More Al_2O_3 like)**	0.1	2.5

Table S3: Parameters varied to create the four JV curves shown in Figure 4C. We note that electronic carrier mobilities were only varied in the 50 nm of perovskite adjacent to the perovskite/ETL interface to simulate the effective reduction in lateral mobility due to electrons having to travel around the insulating Al_2O_3 nanoparticles.

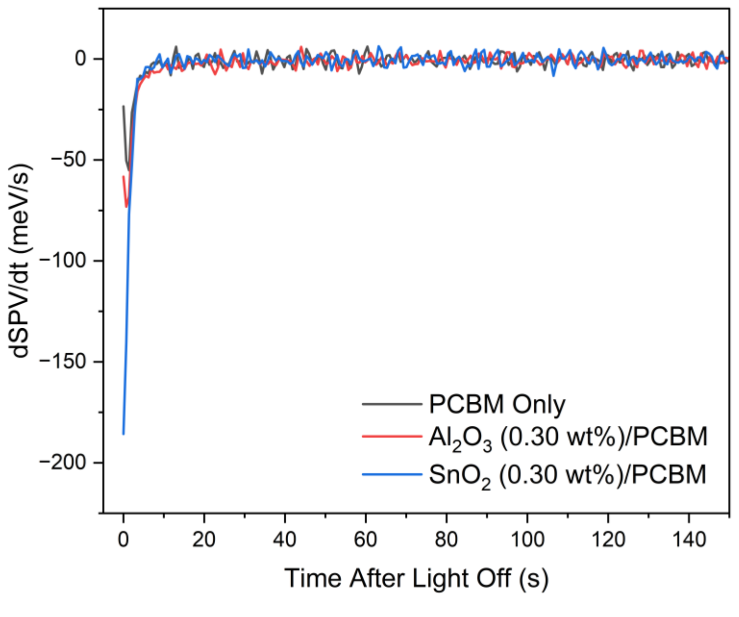

Figure S16: First derivative plots of the SPV decays of reference (black), Al_2O_3 (red), and SnO_2 (blue) nanoparticle modified partial device stacks following illumination. The decay rate at short times is faster for the SnO_2 nanoparticle modified sample than for the Al_2O_3 nanoparticle modified or reference samples. At long times the decay rates for all samples are equivalent.

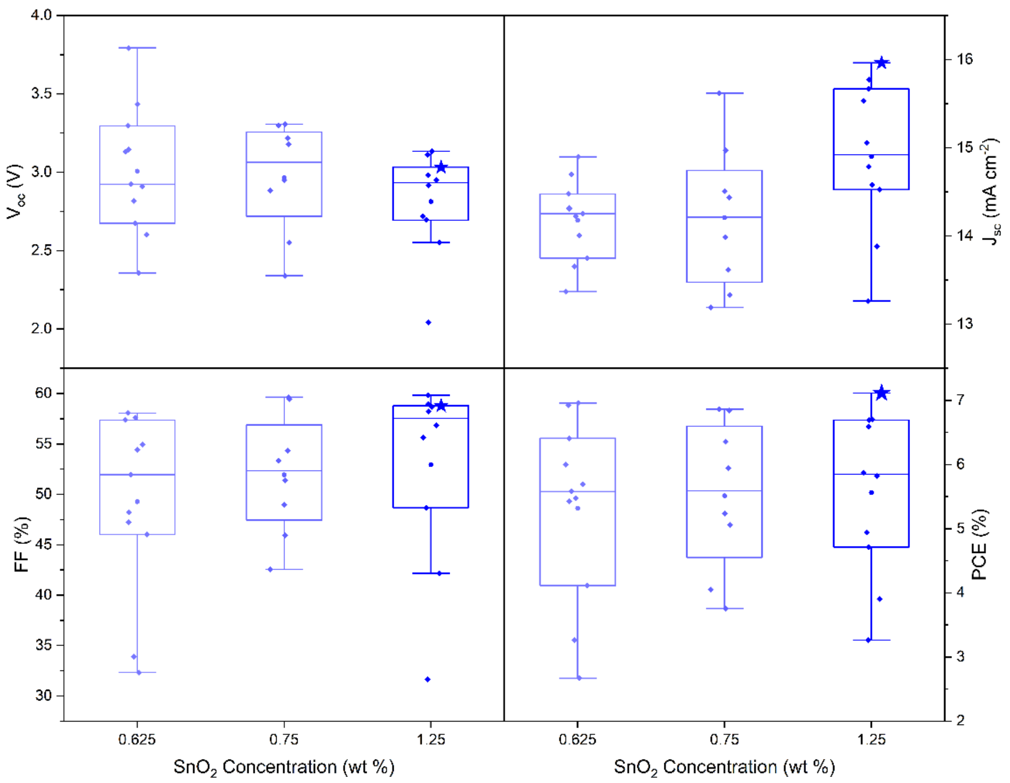

Figure S17: SnO_2 nanoparticle interlayer concentration dependent 1 Sun JV performance parameters of 7.2 cm^2 minimodules. The JV curve of the champion device, marked with a star, is given in Figure 5a of the main text.

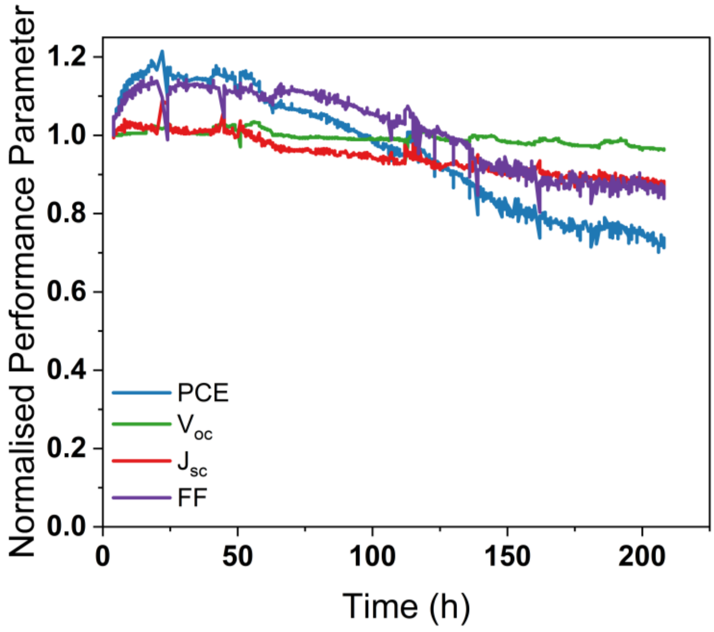

Figure S18: Results of maximum power point tracking (MPPT) stability testing of the champion encapsulated minimodule under 1 Sun conditions in air.

References

(1) Almora, O.; Cabrera, C. I.; Garcia-Cerrillo, J.; Kirchartz, T.; Rau, U.; Brabec, C. J. Quantifying the Absorption Onset in the Quantum Efficiency of Emerging Photovoltaic Devices. *Adv. Energy Mater.* **2021**, *11* (16), 1–9. https://doi.org/10.1002/aenm.202100022.

(2) Ramadan, A. J.; Oliver, R. D. J.; Johnston, M. B.; Snaith, H. J. Methylammonium-Free Wide-Bandgap Metal Halide Perovskites for Tandem Photovoltaics. *Nat. Rev. Mater.* **2023**, *8* (12), 822–838. https://doi.org/10.1038/s41578-023-00610-9.

(3) Chueh, C.-C.; Li, C.-Z.; Jen, A. K.-Y. Recent Progress and Perspective in Solution-Processed Interfacial Materials for Efficient and Stable Polymer and Organometal Perovskite Solar Cells. *Energy Environ. Sci.* **2015**, *8* (4), 1160–1189. https://doi.org/10.1039/C4EE03824J.

(4) Lim, J.; Kober-Czerny, M.; Lin, Y.-H.; Ball, J. M.; Sakai, N.; Duijnstee, E. A.; Hong, M. J.; Labram, J. G.; Wenger, B.; Snaith, H. J. Long-Range Charge Carrier Mobility in Metal Halide Perovskite Thin-Films and Single Crystals via Transient Photo-Conductivity. *Nat. Commun.* **2022**, *13* (1), 4201. https://doi.org/10.1038/s41467-022-31569-w.

(5) Kirchartz, T.; Márquez, J. A.; Stolterfoht, M.; Unold, T. Photoluminescence-Based Characterization of Halide Perovskites for Photovoltaics. *Adv. Energy Mater.* **2020**, *10* (26). https://doi.org/10.1002/aenm.201904134.

(6) Bertoluzzi, L.; Boyd, C. C.; Rolston, N.; Xu, J.; Prasanna, R.; O'Regan, B. C.; McGehee, M. D. Mobile Ion Concentration Measurement and Open-Access Band Diagram Simulation Platform for Halide Perovskite Solar Cells. *Joule* **2020**, *4* (1), 109–127. https://doi.org/10.1016/j.joule.2019.10.003.

(7) Leijtens, T.; Eperon, G. E.; Barker, A. J.; Grancini, G.; Zhang, W.; Ball, J. M.; Kandada, A. R. S.; Snaith, H. J.; Petrozza, A. Carrier Trapping and Recombination: The Role of Defect Physics in Enhancing the Open Circuit Voltage of Metal Halide Perovskite Solar Cells. *Energy Environ. Sci.* **2016**, *9* (11), 3472–3481. https://doi.org/10.1039/C6EE01729K.

(8) Sánchez, J. G.; Cabrera-Espinoza, A.; Martínez-Ferrero, E.; Delgado, J. L.; Palomares, E. Chalcogen-Substituted PCBM Derivatives as Ternary Components in PM6:Y6 Solar Cells. *Mater. Adv.* **2022**, *3* (2), 1071–1078. https://doi.org/10.1039/D1MA00925G.

(9) Mihailetchi, V. D.; van Duren, J. K. J.; Blom, P. W. M.; Hummelen, J. C.; Janssen, R. A. J.; Kroon, J. M.; Rispens, M. T.; Verhees, W. J. H.; Wienk, M. M. Electron Transport in a Methanofullerene. *Adv. Funct. Mater.* **2003**, *13* (1), 43–46. https://doi.org/10.1002/adfm.200390004.

(10) Bässler, H.; Kroh, D.; Schauer, F.; Nádaždy, V.; Köhler, A. Mapping the Density of States Distribution of Organic Semiconductors by Employing Energy Resolved–Electrochemical Impedance Spectroscopy. *Adv. Funct. Mater.* **2021**, *31* (9). https://doi.org/10.1002/adfm.202007738.

(11) Xu, Y.; Yuan, J.; Zhou, S.; Seifrid, M.; Ying, L.; Li, B.; Huang, F.; Bazan, G. C.; Ma, W. Ambient Processable and Stable All-Polymer Organic Solar Cells. *Adv. Funct. Mater.* **2019**, *29* (8). https://doi.org/10.1002/adfm.201806747.
